# The uncertain self: Intolerance of uncertainty moderates the association of both positive and disorganized schizotypal traits with self-concept clarity in a non-clinical sample

**DOI:** 10.1371/journal.pone.0352650

**Published:** 2026-07-02

**Authors:** Alexandra Kaszás, Szabolcs Kéri, Dorottya Bencze, Ágnes Szőllősi, Ágota Vass, Mihály Racsmány, Bertalan Polner

**Affiliations:** 1 Department of Cognitive Science, Budapest University of Technology and Economics, Budapest, Hungary; 2 Sztárai Institute, Sárospatak College, University of Tokaj, Sárospatak, Hungary; 3 Department of Physiology, Albert Szent-Györgyi Medical School, University of Szeged, Szeged, Hungary; 4 Institute of Cognitive Neuroscience and Psychology, HUN-REN Research Centre for Natural Sciences, Budapest, Hungary; 5 Institute of Psychology, University of Szeged, Szeged, Hungary; 6 Department of Psychiatry and Psychotherapy, Semmelweis University, Budapest, Hungary; 7 Institute of Psychology, Eötvös Loránd University, ELTE, Budapest, Hungary; 8 Donders Institute for Brain, Cognition, and Behavior, Nijmegen, Netherlands; University of the West Indies at Saint Augustine, TRINIDAD AND TOBAGO

## Abstract

Our objective was to investigate the relationship between self-concept clarity, intolerance of uncertainty, and schizotypal traits, with a focus on whether intolerance of uncertainty moderates the association between self-concept clarity and positive schizotypy in a non-clinical context. A sample of 315 adults (on average 43 (SD = 12) years, 247 women) completed the Self-Concept Clarity Scale, the Intolerance of Uncertainty scale, and the Schizotypal Personality Questionnaire – Brief Revised. Lower self-concept clarity was significantly associated with higher levels of positive, negative, and disorganized schizotypy, as well as with greater inhibitory intolerance of uncertainty. Importantly, a moderation analysis revealed that intolerance of uncertainty significantly altered the strength of the negative relationship between self-concept clarity and both positive and disorganized schizotypy. Specifically, this association was strongest among individuals with low levels of intolerance of uncertainty. In contrast, at higher levels of intolerance of uncertainty, the negative relationship between self-concept clarity and both positive and disorganized schizotypy was weaker. This pattern suggests that, among individuals with lower tolerance for uncertainty, a better-defined self-concept is more strongly associated with lower levels of positive and disorganized schizotypy. These results underscore the importance of considering both self-structure and uncertainty tolerance in models of schizotypy and psychosis risk. Our findings suggest that self-concept clarity and intolerance of uncertainty are interrelated cognitive factors that synergistically explain variance in schizotypal traits. As intolerance of uncertainty is a potentially modifiable cognitive process, the results have implications for early interventions aimed at reducing psychosis risk by improving self-concept clarity and tolerance for uncertainty. Although the study used a non-clinical sample, it supports a dimensional approach to psychosis and highlights key targets for future research.

## Introduction

The concept of subjectivity—the lived experience of being a self-aware, thinking, feeling individual— and its disorders have become a prominent topic in psychological and psychiatric research, gaining increasing attention from both researchers and the general public. However, schizophrenia—a disorder of subjectivity in which the connection between an individual’s inner experiences and the external world is altered, leading to a distorted perception and interpretation of reality—remains one of the most misunderstood and stigmatized mental illnesses, leaving many open questions for researchers. Studying patients diagnosed with schizophrenia presents significant challenges due to the severity of the illness, poor patient conditions, potential substance abuse, hospitalization, and the side effects of medication [1].

To address these challenges, researchers have developed various animal and human models to investigate psychotic mechanisms. These models fall into two categories: state models, which examine transient conditions, and trait models, which study persistent traits such as high levels of schizotypy in the general population [2]. Schizotypy is commonly conceptualized as a dimensional continuum of schizophrenia-spectrum characteristics, ranging from subclinical traits in the general population to clinical manifestations associated with schizophrenia [3]. This dimensional perspective allows researchers to investigate psychosis-proneness without the confounding effects of chronic illness, medication, or hospitalization commonly present in clinical samples. Studying schizotypal traits in non-clinical populations thus provides a unique opportunity to explore the psychological and cognitive mechanisms underlying psychosis risk in a more controlled and generalizable context. Moreover, it facilitates early identification of vulnerability markers and contributes to our understanding of how certain personality traits may evolve into more severe psychopathological conditions [1].

Like the symptoms of schizophrenia, schizotypal traits can be divided into three dimensions based on factor analysis: positive, negative, and disorganized schizotypy [4]. Positive schizotypy involves an excess of phenomena that should not be present, such as unusual experiences, magical thinking, or ideas of reference [5]. In contrast, negative schizotypy reflects differences in expected functions, such as avolition or an absence of close relationships [6]. Disorganized schizotypy pertains to disturbances in the regulation of thought and behavior [7]. Notably, higher levels of disorganization may predict increased positive and negative traits, and vice versa [6]. As a result, disorganization must be considered to fully differentiate the dimensions of schizotypy.

Self-concept clarity is a central structural feature of the self, defined as the extent to which an individual’s beliefs about themselves are clearly and confidently defined, internally consistent, and stable over time [8]. A healthy self-concept allows an individual to perceive themselves as a coherent and consistent being, maintain a stable yet flexible identity, distinguish themselves from others, and regulate thoughts, emotions, and actions. Consistent with this, self-concept clarity has been shown to correlate with a broad range of psychological outcomes, including resilience, well-being, interpersonal functioning, and emotional adjustment [9,10].

Disturbances in self-structure have been repeatedly linked to schizophrenia-spectrum phenomena. Early theoretical accounts described schizophrenia in terms of fragmentation or disruption of the unity of the self [11]. More recent empirical work suggests that structural aspects of the self-concept (e.g., organization) may be especially relevant in schizophrenia-spectrum conditions. For example, individuals with schizophrenia or first-episode psychosis have been found to show lower self-concept clarity, and reduced self-concept clarity has been associated with more severe symptoms [12,13]. These findings suggest that difficulties in maintaining a coherent and stable self-representation may be relevant not only to clinical schizophrenia but also to subclinical schizophrenia-spectrum traits.

Importantly, self-concept clarity is not specific to schizophrenia-spectrum conditions. In the general population, lower self-concept clarity has been associated with poorer psychological adjustment, lower well-being, and greater emotional distress. Studies examining anxiety-related difficulties have also linked reduced self-concept clarity to higher anxiety and depressive symptoms. [14] Thus, self-concept clarity may represent a broader psychological factor that is relevant across both clinical and non-clinical forms of psychopathology [9,10].

Another construct that may be relevant to self-related difficulties and schizotypal traits is intolerance of uncertainty. Intolerance of uncertainty refers to a dispositional tendency to perceive ambiguous, unpredictable, or insufficiently defined situations as aversive [15]. Contemporary models conceptualize intolerance of uncertainty as a transdiagnostic mechanism involved in fear of the unknown, anxiety, and related forms of psychopathology [16]. In this framework, uncertainty may become a central source of threat, motivating attempts to reduce ambiguity and restore predictability.

Intolerance of uncertainty is not considered a unitary construct. Factor-analytic work has supported a distinction between two related but separable dimensions: prospective intolerance of uncertainty and inhibitory intolerance of uncertainty [17]. Prospective intolerance of uncertainty reflects a desire for predictability and an active orientation toward obtaining certainty, whereas inhibitory intolerance of uncertainty refers to the tendency for uncertainty to interfere with action, decision-making, or goal-directed behavior. This distinction may be particularly relevant in schizotypy, where uncertainty may be managed either through heightened efforts to impose predictability and meaning or through uncertainty-related inhibition and reduced perceived control.

Intolerance of uncertainty has also been linked to psychosis-spectrum phenomena. In clinical samples, higher intolerance of uncertainty has been associated with more severe positive, negative, and general symptoms [18], and studies suggest that delusional thinking and distress related to psychosis may be connected to difficulties tolerating uncertainty [19,20] One possible explanation is that rigid or unusual beliefs may temporarily reduce uncertainty by providing a sense of meaning, predictability, or control. These findings suggest that intolerance of uncertainty may be relevant to schizotypal traits, particularly positive and disorganized features involving unusual interpretations, anomalous experiences, or disturbances in cognitive organization [21].

Only a limited number of studies have directly examined the association between self-concept clarity and intolerance of uncertainty. In general-population and anxiety-related samples, lower self-concept clarity has been associated with higher intolerance of uncertainty, anxiety, and depressive symptoms [14,22]. Butzer and Kuiper [14], in a study with 166 participants, found a negative association between these two factors in the general population, while both of them were related to anxiety and depression. Kusec et al. [22] further explored this connection in the context of generalized anxiety disorder (GAD). Their findings showed that individuals with high GAD symptoms exhibited two distinguishing traits: increased intolerance of uncertainty and reduced self-concept clarity.

These findings suggest that an unclear or unstable self-concept may be linked to greater difficulty tolerating ambiguity and unpredictability. However, previous studies do not establish whether intolerance of uncertainty functions as a mediator or moderator in the association between self-concept clarity and schizotypal traits.

In the present study, we conceptualized intolerance of uncertainty as a moderator of the association between self-concept clarity and schizotypal traits. The rationale behind our hypothesis was that intolerance of uncertainty may shape the extent to which low self-concept clarity is associated with schizotypal features. Specifically, individuals who experience uncertainty as more aversive or disabling may differ in how an unclear self-concept relates to unusual experiences, unusual beliefs, or disorganized cognition. People with high intolerance of uncertainty are more sensitive to violations of internal coherence (such as an unclear self-concept), which could amplify unusual schizotypal experiences and magical thinking [23]. Thus, our primary question was not whether intolerance of uncertainty transmits the effect of self-concept clarity on schizotypy, but whether the strength of the self-concept clarity – schizotypy association varies across levels of intolerance of uncertainty.

The present study aimed to examine the associations among self-concept clarity, intolerance of uncertainty, and schizotypal traits in a non-clinical sample. In line with previous findings, we hypothesized that lower self-concept clarity would be associated with higher positive, negative, and disorganized schizotypy, and with higher intolerance of uncertainty. We further hypothesized that intolerance of uncertainty would moderate the association between self-concept clarity and schizotypal traits. Given evidence that intolerance of uncertainty consists of prospective and inhibitory dimensions, we examined these two facets separately. Because previous studies have not directly tested these interaction effects in the context of schizotypy, analyses involving the two intolerance of uncertainty dimensions were considered partly exploratory.

## Method

### Participants

Participants aged 18 and over were recruited between September 4 and December 31, 2022, via announcements posted on the research team’s Facebook page. The study targeted a non-clinical community sample, and any adult was eligible to participate. Individuals under the age of 18 or those who self-reported a current or past psychiatric diagnosis were excluded; an automated screening mechanism prevented ineligible respondents from completing the questionnaire.

Here, we analyse data from a study chiefly designed to estimate the associations between schizotypal traits and memory performance (will be reported in a separate study). In the context of that research question, based on a power analysis (the univariate association between schizotypy and visual memory alterations is expected to be “small”: r = 0.2, alpha = 0.05, beta = 0.1 [24]), the minimum required sample size was determined to be 259 participants. Because the association between schizotypy and other self-reported traits is expected to be in general higher than that of schizotypy and memory due to common method variance, the above estimation of 90% (1 – beta * 100) provides a lower bound for the statistical power obtained for the bivariate associations in this study. Recruitment was carried out using a convenience sampling method, with participants recruited through personal networks and paid advertisements (without any ad targeting regarding participants’ personal interests or demographic characteristics). Participation was voluntary, and anonymity was ensured. Before beginning the survey, participants received detailed information about the study. They were required to explicitly indicate their informed consent by checking boxes confirming that they had read the information and agreed to participate. A password system was implemented to prevent duplicate entries. The study adhered to the ethical principles outlined in the Helsinki Declaration and was approved by the United Ethical Review Committee for Research in Psychology, Hungary (2016/032, 2019/29).

Initially, 320 participants completed the study. One participant was excluded for providing identical responses to all items on the Self-Concept Clarity Scale, including reversed items. An additional four participants were excluded for failing to complete the Schizotypy Personality Questionnaire. As a result, the final sample consisted of 315 participants (self-reported sex: 67 men, 247 women, and 1 non-binary). The average age of the participants was 43 years, with a range from 18 to 74 years (SD = 11.9 years). In terms of education, one-third (32%) of the participants held a Bachelor’s degree, another third (32%) had a higher education degree, and the remaining third (36%) had lower education. Twenty-eight percent of the respondents reported being currently enrolled in educational studies. Geographically, 32% of the sample resided in the capital, 54% lived in other cities or towns, and the remaining participants (14%) lived in settlements with fewer than 1,000 inhabitants. In terms of marital status, 59% of participants were married or in a relationship, 28% were single, and 13% were divorced or widowed.

### Questionnaires

All questionnaires were administered using previously validated Hungarian versions. For each measure, we cite both the original publication and the Hungarian validation study, respectively.

Schizotypy was assessed using the Schizotypal Personality Questionnaire – Brief Revised (SPQ-BR; [25,26]). The 32 items of the questionnaire can be summed along the dimensions of positive (14 items), negative/interpersonal (10 items), and disorganized (8 items) schizotypy. The subscale scores were all highly reliable in the sample, *SPQ positive: α = 0.850, 95% CI [0.826–0.875]; SPQ negative: α = 0.864, 95% CI [0.841–0.887]; SPQ disorganized: α = 0.817, 95% CI [0.782–0.852]*). The Hungarian validation study reported good internal consistency for the scales (*Social anxiety:* α = *0.88; Eccentric behavior:* α = *0.87; Odd speech:* α = *0.84; Magical thinking:* α = *0.84; No close friends/Constricted Affect:* α = *0.80; Ideas of Reference/Suspiciousness:* α = *0.73; Unusual experiences:* α = *0.71;*). Participants were asked to rate their agreement with each statement on a 5-point Likert scale ranging from 1 to 5.

Self-concept clarity was measured using the Self-Concept Clarity Scale (SCCS; [8,27]), which assesses the clarity and strength of individuals’ beliefs about themselves using 12 items (*α = 0.896, 95% CI [0.877–0.915]*). The Hungarian validation study reported good internal consistency for the scale (*α = 0.86*). Participants rated each item on a scale from 1 (=disagree) to 5 (=agree).

Intolerance of uncertainty was assessed using the short version of the Intolerance of Uncertainty Scale (IUS-12; [28,29]), which measures individuals’ ability to tolerate the occurrence and unpredictability of negative events. The 12 items are divided into two subscales: prospective and inhibitory anxiety. The prospective subscale (IUS PRO) includes 7 items about the fear of future events, while the inhibitory subscale (IUS INH) of 5 items inquires about questions about the incapacity to act when facing uncertainty (*IUS PRO: α = 0.780, 95% CI [0.738–0.823]; IUS INH: α = 0.729, 95% CI [0.675–0.784]; IUS total: α = 0.846, 95% CI [0.816–0.876]*). The Hungarian validation study reported good internal consistency for the scales (*IUS PRO: ⍵ = 0.82; IUS INH: ⍵ = 0.84*). Participants rated each statement on a scale from 1 (=strongly disagree) to 5 (=strongly agree), depending on the statement they agreed with the most.

Based on Cronbach’s α values, all subscales met the 0.7 threshold, indicating good internal consistency and reliability.

### Procedure

The test materials were administered to participants using the open-source software *formr* [30], where the data were also stored. This platform provides researchers with an innovative tool for designing questionnaires and tests, distributing them to participants, and enhancing reproducibility. We utilized the software to monitor data collection and track participant feedback. Questionnaires were administered in a fixed order: SCCS, IUS-12, and SPQ-BR.

### Statistical analysis

Our research questions were tested using data analysis in JASP (version 0.19.3.0) [31], and data visualization was conducted using the R programming language. The Shapiro-Wilk test indicated that normality was violated for all investigated variables, which was accounted for in the analysis. Additionally, variable distributions were assessed visually.

The relationships between schizotypy dimensions and self-concept clarity, as well as between self-concept clarity and intolerance of uncertainty, were examined using non-parametric correlation (rho). The potential moderating role of intolerance of uncertainty in the relationship between the different dimensions of schizotypy and self-concept clarity was assessed through linear regression models. These models were adjusted for age and sex, as previous research indicates associations between these factors and schizotypy [32] as well as self-concept clarity [33,34]. A 95% confidence interval was calculated. To account for multiple testing across the six moderation models, p-values for the interaction terms were adjusted using the Benjamini–Hochberg false discovery rate procedure.

## Results

The means and standard deviations of the used scales can be found in Table 1.

**Table 1:** Descriptive statistics for SPQ dimensions, self-concept clarity, and dimensions of intolerance of uncertainty.

Descriptive	SPQ POS	SPQ NEG	SPQ DIS	SCCS	IUS PRO	IUS INH	IUS total
Mean	33.21	25.33	21.61	44.87	21.12	13.70	34.82
St. Deviation	9.675	8.097	6.127	9.031	4.689	3.526	7.449

*Note.* N = 315. SPQ POS = Schizotypy Personality Questionnaire Positive Dimension. SPQ NEG = Schizotypy Personality Questionnaire Negative Dimension. SPQ DIS = Schizotypy Personality Questionnaire Disorganized Dimension. SCCS = Self-Concept Clarity. IUS PROS = Prospective intolerance of uncertainty. IUS INH = Inhibitory intolerance of uncertainty. IUS total = Total intolerance of uncertainty score.

**Fig 1:**
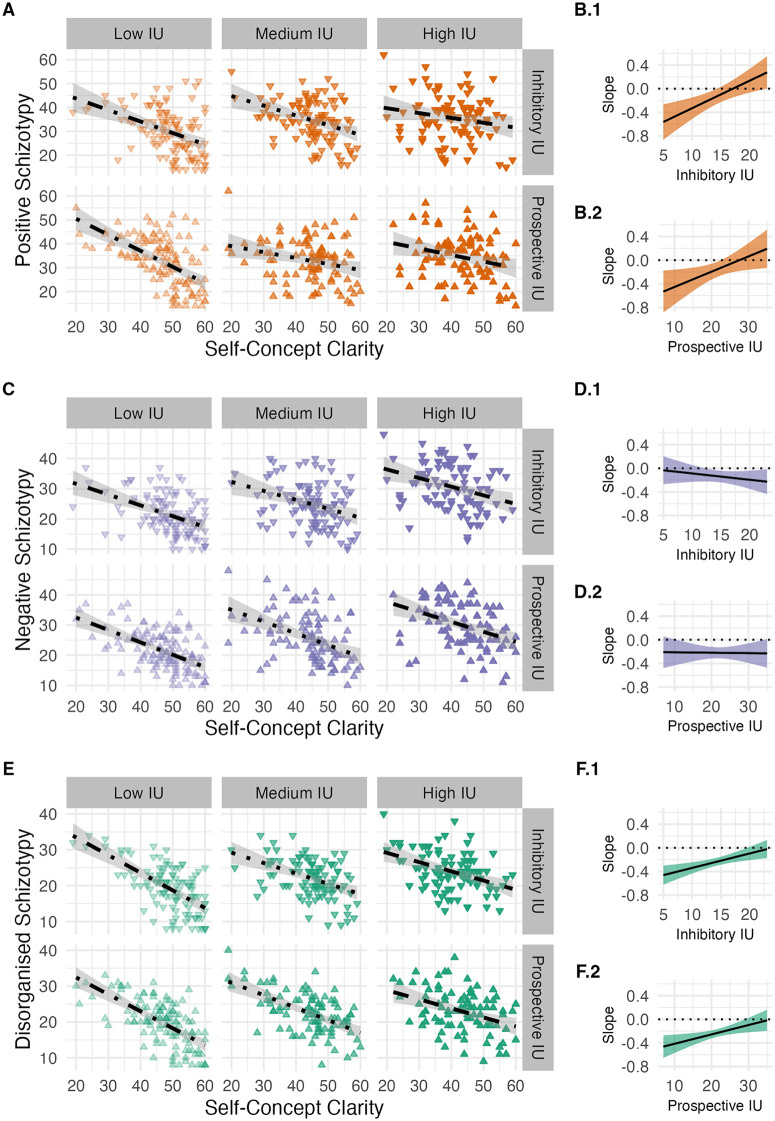
Association of self-concept clarity with schizotypy dimensions as a function of intolerance of uncertainty (IU). The ribbons indicate 95% confidence intervals. **A:** the association between self-concept clarity and positive schizotypy varied across low, medium, and high IU subgroups. **B:** the estimated slope coefficient between self-concept clarity and positive schizotypy as a function of IU. **C:** association between self-concept clarity and negative schizotypy did not significantly vary across low, medium, and high IU subgroups. **D:** estimated slope coefficient between self-concept clarity and negative schizotypy as a function of IU. **E:** the association between self-concept clarity and disorganised schizotypy varied across low, medium, and high IU subgroups. **F:** estimated slope coefficient between self-concept clarity and disorganised schizotypy as a function of IU. Low, medium, and high levels of Intolerance of Uncertainty were based on tertile splits for visualisation (panels A, C, **E)**. Slope estimates were extracted from a linear model where a dimension of schizotypy was regressed on self-concept clarity, intolerance of uncertainty, and their interaction; the models were adjusted for age, sex, and the other dimensions of schizotypy (panels B, D, **F)**.

We examined the relationship between self-concept clarity and the three dimensions of schizotypy using non-parametric correlation (rho) with partialization. Confidence intervals were estimated based on 5000 bootstrap samples. This analysis aimed to establish a foundation for our additional research questions. To account for potential confounders, we controlled for sex and age. As shown in Fig 1 and Table 2, the results indicated that self-concept clarity was negatively associated with all three schizotypy dimensions: **positive** (*ρ = −0.386, p < 0.001, 95% CI [−0.485, −0.280]*), **negative** (*ρ = −0.482, p < 0.001, 95% CI [−0.569, −0.387]*), and **disorganized** (*ρ = −0.528, p < 0.001, 95% CI [−0.609, −0.437]*). All associations were moderately negative, suggesting that higher levels of schizotypy are associated with lower self-concept clarity, and vice versa. After Benjamini–Hochberg correction, all correlations remained significant.

**Table 2:** Non-parametric correlations (rho) between self-concept clarity, SPQ dimensions, and intolerance of uncertainty.

Variable	1	2	3	4	5	6
SCCS	–					
SPQ Positive	−0.386***	–				
SPQ Negative	−0.482***	0.316***	–			
SPQ Disorganized	−0.528***	0.443***	0.464***	–		
IUS PROS	−0.185**	0.068	0.455***	0.232***	–	
IUS INH	−0.440***	0.299***	0.488***	0.305***	0.629***	–
IUS total	−0.318***	0.172**	0.518***	0.288***	0.931***	0.858***

*Note.* N = 315. SPQ = Schizotypy Personality Questionnaire. SCCS = Self-Concept Clarity. IUS PROS = Prospective intolerance of uncertainty. IUS INH = Inhibitory intolerance of uncertainty. IUS total = Total intolerance of uncertainty score. Non-parametric correlations (rho) reported, controlling for age and gender. * < .05, *** p < .001. For IUS PROS and positive schizotypy, p = .229. *p*-values were adjusted for multiple comparisons using the Benjamini–Hochberg procedure. The significance levels reported in the table are based on the adjusted *p*-values.

Next, we examined the relationship between self-concept clarity and the two dimensions of intolerance of uncertainty using non-parametric correlation (rho), controlling for sex and age. Self-concept clarity exhibited a weak negative association with **prospective intolerance of uncertainty** (*ρ = −0.185, p < 0.001, 95% CI [−0.291, −0.071]*) and a moderate negative association with **inhibitory intolerance of uncertainty** (*ρ = −0.440, p < 0.001, 95% CI [−0.529, −0.344]*). Self-concept clarity also showed a moderate negative association with the **total intolerance of uncertainty score (***ρ = −0.318, p < 0.001, 95% CI [−0.413, −0.210])*. The results are shown in Table 2.

To examine moderation effects, six linear regression analyses were conducted, with the three dimensions of schizotypy serving as dependent variables. For each schizotypy dimension, two separate regression models were estimated. In both models, self-concept clarity was included as a predictor; however, the dimensions of intolerance of uncertainty were entered separately: one model included the prospective dimension, while the other included the inhibitory dimension. An interaction term between self-concept clarity and the respective intolerance of uncertainty dimension was added in each model to test the moderation effect. Age and sex were entered as control variables. Additionally, when a given schizotypy dimension was treated as the dependent variable, the remaining two schizotypy dimensions were controlled for (e.g., in models predicting positive schizotypy, negative and disorganized schizotypy were included as covariates). Although we have previously argued why we decided to analyze the dimensions of the IUS instead of simply using the total score of the scale, we have included models with the total score as well for better comparisons to other research data for possible metanalytic approaches. For an overview of the moderation analyses, see Fig 1.

### Positive schizotypy

We found a significant interaction between self-concept clarity and both dimensions of intolerance of uncertainty. For the inhibitory dimension, the interaction term yielded *Beta = 0.047, SE = 0.014, Standardized Beta = 0.758, t = 3.228, p = 0.001*, while for the prospective dimension, it was *Beta = 0.026, SE = 0.011, Standardized Beta = 0.703, t = 2.254, p = 0.025*. These findings suggest that, contrary to our hypothesis, higher levels of intolerance of uncertainty dampen the association of self-concept clarity with positive schizotypy. Conversely, when intolerance of uncertainty is lower, the association of self-concept clarity with positive schizotypy is stronger. Fig 1A and 1B illustrate how different levels of intolerance of uncertainty moderate this relationship, and the models are summarised in Tables 3 and 4. Table 5 shows the model including the total IU score.

**Table 3:** Linear regression results for positive schizotypy with prospective IU as moderator.

Predictor	B	SE B	ß	t	p
Intercept	49.481	13.101		3.777	<.001
SCCS	−0.712	0.258	−0.665	−2.763	0.006
IUS PROS	−1.319	0.533	−0.639	−2.476	0.014
Disorganized	0.553	0.100	0.350	5.542	<.001
Negative	0.131	0.076	0.109	1.721	0.086
Age	0.070	0.040	0.086	1.750	0.081
Sex	0.540	1.132	0.023	0.477	0.634
SCCS x IUS PROS	0.026	0.011	0.703	2.254	0.025

*Note.* N = 315. SCCS = Self-Concept Clarity. IUS PROS = Prospective Intolerance of Uncertainty. Disorganized = Disorganized dimension of schizotypy. Negative = Negative dimension of schizotypy. B = unstandardized coefficients; SE B = standard error of B; ß = standardized coefficient.

**Table 4:** Linear regression results for positive schizotypy with inhibitory IU as moderator.

Predictor	B	SE B	ß	t	p
Intercept	48.236	11.463		4.208	<.001
SCCS	−0.796	0.219	−0.743	−3.627	<.001
IUS INH	−1.680	0.662	−0.612	−2.536	0.012
Disorganized	0.509	0.099	0.322	5.148	<.001
Negative	0.034	0.074	0.029	0.467	0.641
Age	0.061	0.039	0.075	1.541	0.124
Sex	0.665	1.115	0.029	0.596	0.552
SCCS x IUS INH	0.047	0.014	0.758	3.228	0.001

*Note.* N = 315. SCCS = Self-Concept Clarity. IUS INH = Inhibitory Intolerance of Uncertainty. Disorganized = Disorganized dimension of schizotypy. Negative = Negative dimension of schizotypy. B = unstandardized coefficients; SE B = standard error of B; ß = standardized coefficient.

**Table 5:** Linear regression results for positive schizotypy with the total IU score as moderator.

Predictor	B	SE B	ß	t	p
Intercept	56.823	13.660		4.160	<.001
SCCS	−0.916	0.269	−0.855	−3.405	<.001
IUS total	−0.917	0.332	−0.706	−2.767	0.006
Disorganized	0.515	0.100	0.326	5.134	<.001
Negative	0.085	0.077	0.071	1.100	0.272
Age	0.066	0.040	0.082	1.666	0.097
Sex	0.560	1.130	0.024	0.496	0.620
SCCS x IUS total	0.021	0.007	0.842	2.912	0.004

*Note.* N = 315. SCCS = Self-Concept Clarity. IUS total = Total Intolerance of Uncertainty score. Disorganized = Disorganized dimension of schizotypy. Negative = Negative dimension of schizotypy. B = unstandardized coefficients; SE B = standard error of B; ß = standardized coefficient.

The first model yielded the following values: R = 0.526; R² = 0.277; Adjusted R² = 0.263; RMSE = 8.307. The second model produced comparable values: R = 0.557; R² = 0.311; Adjusted R² = 0.295; RMSE = 8.123. The third model, which included the IUS total score, yielded R = 0.541, R² = 0.293, adjusted R² = 0.277, and RMSE = 8.229. Overall, the R values indicate a moderately strong association between the predictors and the positive dimension of schizotypy across all three models. Collectively, the models explain approximately 28–31% of the variance in positive schizotypy, which is a substantial proportion. Additionally, the close alignment of the adjusted R² values with R² suggests that the predictors contribute meaningfully to explaining positive schizotypy and that the models are not overfitted.

Regarding the control variables, age and sex did not exhibit a significant effect in either model. However, the disorganized dimension demonstrated a significant influence (**prospective model**: *Beta = 0.553, SE = 0.100, Standardized Beta = 0.350, t = 5.542, p < 0.001*; **inhibitory model**: *Beta = 0.509, SE = 0.099, Standardized Beta = 0.322, t = 5.148, p < 0.001;* **model with the total scores**: *Beta = 0.515, SE = 0.100, Standardized Beta = 0.326, t = 5.134, p < 0.001*). This was expected, as higher levels of disorganization may predict increased positive and negative traits, and vice versa [6]. However, the interaction remained significant even when controlling for this variable, indicating that the results cannot be explained solely by the central role of disorganisation.

### Negative schizotypy

No significant interaction effects emerged between self-concept clarity and either dimension of intolerance of uncertainty. Specifically, in the model including the inhibitory dimension of intolerance of uncertainty, the interaction term was not significant (*Beta = −0.011, SE = 0.011, Standardized Beta = −0.209, t = −0.949, p = 0.343*). Similarly, the interaction between self-concept clarity and the prospective dimension did not reach statistical significance (*Beta = 0.073, SE = 0.042, Standardized Beta = 0.087, t = 1.727, p = 0.085*.). A third model including the total score of intolerance of uncertainty yielded the same pattern of results, indicating no significant interaction effect (*Beta = −0.002, SE = 0.005, Standardized Beta = −0.099, t = −0.384, p = 0.702*.). The associations are shown in Fig 1C, and the lack of moderation is visualized in Fig 1D, while the regression models are summarized in Tables 6–8.

**Table 6:** Linear regression results for negative schizotypy with prospective IU as moderator.

Predictor	B	SE B	ß	t	p
Intercept	15.816	3.954		4.000	<.001
SCCS	−0.221	0.048	−0.247	−4.657	<.001
IUS PROS	0.607	0.077	0.351	7.856	<.001
Disorganized	0.297	0.075	0.225	3.946	<.001
Positive	0.607	0.077	0.351	7.856	<.001
Age	−0.063	0.030	−0.093	−2.126	0.034
Sex	0.286	0.845	0.015	0.338	0.735
SCCS x IUS PROS	0.073	0.042	0.087	1.727	0.085

*Note.* N = 315. SCCS = Self-Concept Clarity. IUS PROS = Prospective Intolerance of Uncertainty. Disorganized = Disorganized dimension of schizotypy. Positive = Positive dimension of schizotypy. B = unstandardized coefficients; SE B = standard error of B; ß = standardized coefficient.

**Table 7:** Linear regression results for negative schizotypy with inhibitory IU as moderator.

Predictor	B	SE B	ß	t	p
Intercept	6.563	9.103		0.721	0.471
SCCS	0.020	0.173	0.023	0.117	0.907
IUS INH	1.267	0.512	0.552	2.474	0.014
Disorganized	0.394	0.076	0.298	5.164	<.001
Positive	0.021	0.044	0.025	0.467	0.641
Age	−0.068	0.030	−0.100	−2.247	0.025
Sex	0.390	0.862	0.020	0.453	0.651
SCCS x IUS INH	−0.011	0.011	−0.209	−0.949	0.343

*Note.* N = 315. SCCS = Self-Concept Clarity. IUS INH = Inhibitory Intolerance of Uncertainty. Disorganized = Disorganized dimension of schizotypy. Positive = Positive dimension of schizotypy. B = unstandardized coefficients; SE B = standard error of B; ß = standardized coefficient.

**Table 8:** Linear regression results for negative schizotypy with the total IU score as moderator.

Predictor	B	SE B	ß	t	p
Intercept	8.765	10.390		0.844	0.400
SCCS	−0.098	0.203	−0.109	−0.481	0.631
IUS total	0.511	0.247	0.470	2.071	0.039
Disorganized	0.321	0.075	0.243	4.261	<.001
Positive	0.046	0.042	0.055	1.100	0.272
Age	−0.066	0.029	−0.097	−2.233	0.026
Sex	0.390	0.837	0.020	0.466	0.641
SCCS x IUS total	−0.002	0.005	−0.099	−0.384	0.702

*Note.* N = 315. SCCS = Self-Concept Clarity. IUS total = Total Intolerance of Uncertainty score. Disorganized = Disorganized dimension of schizotypy. Positive = Positive dimension of schizotypy. B = unstandardized coefficients; SE B = standard error of B; ß = standardized coefficient.

In the first regression model, the overall fit indices indicated a good model fit (R = 0.657, R² = 0.432, adjusted R² = 0.421, RMSE = 6.162). Comparable results were obtained for the second model (R = 0.642, R² = 0.412, adjusted R² = 0.399, RMSE = 6.277), as well as for the third model including the total score of intolerance of uncertainty, which showed a similar pattern of fit indices (R = 0.668, R² = 0.446, adjusted R² = 0.433, RMSE = 6.096). In all cases, the magnitude of the R coefficients reflects a strong association between the set of predictors and the negative schizotypy dimension. Together, the three models account for a substantial proportion of variance in negative schizotypy (approximately 41–44%). Moreover, the minimal difference between R² and adjusted R² across models indicates stable explanatory power and suggests that model complexity did not result in overfitting.

These findings suggest that the associations between self-concept clarity and negative schizotypy are not contingent upon levels of intolerance of uncertainty. Rather, self-concept clarity and intolerance of uncertainty appear to contribute independently to negative schizotypy, indicating additive rather than interactive effects within the examined models.

### Disorganized schizotypy

In contrast to the models predicting negative schizotypy, significant interaction effects emerged between self-concept clarity and both dimensions of intolerance of uncertainty. Specifically, the interaction with the inhibitory dimension was significant (Beta = 0.024, SE = 0.008, standardized Beta = 0.628, t = 3.054, p = 0.002), as was the interaction involving the prospective dimension (Beta = 0.016, SE = 0.006, standardized Beta = 0.682, t = 2.542, p = 0.011). The third model, which included the total score of intolerance of uncertainty, yielded a similar pattern of results, also indicating a significant interaction effect (Beta = 0.013, SE = 0.004, standardized Beta = 0.794, t = 3.196, p = 0.002).

These interaction effects indicate that the relationship between self-concept clarity and disorganized schizotypy is moderated by intolerance of uncertainty. Specifically, higher levels of both inhibitory and prospective intolerance of uncertainty dampen the association between self-concept clarity and disorganized schizotypy. In contrast, the negative association between self-concept clarity and disorganized schizotypy becomes more pronounced at lower levels of intolerance of uncertainty, a pattern shown in Fig 1E and 1F, and regression model summaries are presented in Tables 9–11.

**Table 10:** Linear regression results for disorganized schizotypy with inhibitory IU as moderator.

Predictor	B	SE B	ß	t	p
Intercept	37.041	6.182		5.992	<.001
SCCS	−0.584	0.120	−0.861	−4.882	<.001
IUS INH	−1.141	0.365	−0.656	−3.124	0.002
Negative	0.203	0.039	0.268	5.164	<.001
Positive	0.156	0.030	0.247	5.148	<.001
Age	0.025	0.022	0.049	1.150	0.251
Sex	0.156	0.618	0.011	0.252	0.801
SCCS x IUS INH	0.024	0.008	0.628	3.054	0.002

*Note.* N = 315. SCCS = Self-Concept Clarity. IUS INH = Inhibitory Intolerance of Uncertainty. Negative = Negative dimension of schizotypy. Positive = Positive dimension of schizotypy. B = unstandardized coefficients; SE B = standard error of B; ß = standardized coefficient.

**Table 11:** Linear regression results for disorganized schizotypy with the total IU score as moderator.

Predictor	B	SE B	ß	t	p
Intercept	40.313	7.314		5.512	<.001
SCCS	−0.677	0.145	−0.998	−4.681	<.001
IUS total	−0.530	0.181	−0.644	−2.929	0.004
Negative	0.174	0.041	0.230	4.261	<.001
Positive	0.154	0.030	0.242	5.134	<.001
Age	0.022	0.022	0.042	0.990	0.323
Sex	0.203	0.617	0.014	0.328	0.743
SCCS x IUS total	0.013	0.004	0.794	3.196	0.002

*Note.* N = 315. SCCS = Self-Concept Clarity. IUS total = Total Intolerance of Uncertainty score. Negative = Negative dimension of schizotypy. Positive = Positive dimension of schizotypy. B = unstandardized coefficients; SE B = standard error of B; ß = standardized coefficient.

**Table 9:** Linear regression results for disorganized schizotypy with prospective IU as moderator.

Predictor	B	SE B	ß	t	p
Intercept	34.650	7.042		4.920	<.001
SCCS	−0.573	0.139	−0.845	−4.136	<.001
IUS PROS	−0.620	0.292	−0.474	−2.126	0.034
Negative	0.159	0.041	0.210	3.909	<.001
Positive	0.165	0.030	0.260	5.542	<.001
Age	0.021	0.022	0.040	0.954	0.341
Sex	0.251	0.618	0.017	0.407	0.684
SCCS x IUS PROS	0.016	0.006	0.682	2.542	0.011

*Note.* N = 315. SCCS = Self-Concept Clarity. IUS PROS = Prospective Intolerance of Uncertainty. Negative = Negative dimension of schizotypy. Positive = Positive dimension of schizotypy. B = unstandardized coefficients; SE B = standard error of B; ß = standardized coefficient.

The fit indices of both regression models indicated strong and nearly identical model performance (Model 1: R = 0.687; R² = 0.472; Adjusted R² = 0.460; RMSE = 4.504; Model 2: R = 0.687; R² = 0.472; Adjusted R² = 0.460; RMSE = 4.502; Model 3: R = 0.689; R² = 0.474; Adjusted R² = 0.462; RMSE = 4.494). In each case, the predictors accounted for a substantial proportion of variance in disorganized schizotypy, explaining approximately 47% of the outcome variance. The minimal discrepancy between R² and adjusted R² further suggests that the explanatory power of the models was not driven by overfitting.

With respect to the control variables, neither age nor sex emerged as significant predictors in either regression model. In contrast, both the negative and positive dimensions of schizotypy showed significant associations with disorganized schizotypy across models. In the prospective intolerance of uncertainty model, the negative dimension was a significant predictor (B = 0.159, SE = 0.041, β = 0.210, t = 3.909, p < .001), as was the positive dimension (B = 0.165, SE = 0.030, β = 0.260, t = 5.542, p < .001). A comparable pattern was observed in the inhibitory model, with significant effects for the negative dimension (B = 0.203, SE = 0.039, β = 0.268, t = 5.164, p < .001) and the positive dimension (B = 0.156, SE = 0.030, β = 0.247, t = 5.148, p < .001).

These associations are consistent with prior findings suggesting substantial overlap among schizotypy dimensions, whereby elevated disorganization is accompanied by increased positive and negative schizotypal traits, and vice versa [6]. Importantly, the interaction effects between self-concept clarity and intolerance of uncertainty remained statistically significant after controlling for these dimensions, indicating that the observed moderation effects cannot be attributed solely to the shared variance associated with disorganization.

### Supplementary analysis

In light of a recent systematic review linking intolerance of uncertainty to paranoia-related symptoms in non-clinical samples [35], we conducted supplementary linear regression analyses to clarify the associations between the relevant subscales of positive schizotypy (ideas of reference and suspiciousness) and the other variables under study. In these models, the two subscales were examined separately as dependent variables, resulting in four regression models examining ideas of reference and suspiciousness in relation to the prospective and inhibitory dimensions of intolerance of uncertainty. As in the main analyses, age, sex, and the negative and disorganized schizotypy dimensions were included as covariates.

Across the supplementary models, similar patterns emerged for the two schizotypy subscales examined. Both ideas of reference and suspiciousness were primarily associated with the other schizotypy dimensions: the negative and disorganized dimensions showed significant positive associations, whereas self-concept clarity, prospective and inhibitory intolerance of uncertainty, and their interaction were not significant predictors in either model. Detailed results of these supplementary regression analyses are reported in the Supporting Information section.

## Discussion

The present study provides novel insights into the interplay between self-concept clarity, intolerance of uncertainty, and schizotypal traits. Consistent with prior research, self-concept clarity was negatively associated with all three dimensions of schizotypy, indicating that individuals with a less coherent and stable self-concept tend to report elevated positive, negative, and disorganized schizotypal features. These findings align with theoretical and empirical work emphasizing self-disturbances as a core characteristic of schizotypy and related psychopathological conditions [9,12,13,36], and further support the relevance of self-concept clarity as an important factor in understanding schizotypal personality traits.

In addition, self-concept clarity was negatively related to intolerance of uncertainty, suggesting that individuals with a less stable self-concept may be particularly vulnerable to difficulties in coping with uncertain situations. This pattern is consistent with models proposing that uncertainty is especially distressing for individuals with impaired self-structure, potentially contributing to heightened cognitive and emotional strain [8,37].

At bivariate level, the absence of a significant association between prospective intolerance of uncertainty and positive schizotypy may reflect the distinct psychological processes underlying these constructs. Prospective intolerance of uncertainty captures an active, future-oriented cognitive engagement with uncertainty, whereas positive schizotypy predominantly involves perceptual anomalies and unusual belief systems that may not rely on anticipatory uncertainty processing. Importantly, the lack of a zero-order correlation does not preclude conditional effects, as moderation can occur independently of direct associations between variables.

The moderation analyses revealed that intolerance of uncertainty altered the strength of the association between self-concept clarity and both positive and disorganized schizotypy. Specifically, the pattern of results suggests that when intolerance of uncertainty is relatively low, self-concept clarity shows a stronger negative association with positive and disorganized schizotypal traits. In contrast, at higher levels of intolerance of uncertainty, this association appears attenuated, indicating that elevated uncertainty intolerance may be linked to increased levels of positive and disorganized schizotypy regardless of self-concept clarity. One possible interpretation is that lower intolerance of uncertainty allows greater behavioral and cognitive flexibility, thereby enabling self-concept clarity to play a more decisive role in shaping schizotypal expression, whereas high intolerance of uncertainty may contribute to rigidity, avoidance, and emotional overcontrol that override self-structural influences.

Notably, no significant interaction effects emerged for negative schizotypy. This finding is consistent with prior work conceptualizing negative schizotypy as a more stable, trait-like dimension characterized by reduced affective reactivity and lower motivational engagement, which may render it less sensitive to contextual or cognitive moderators such as intolerance of uncertainty [38,39].

From a broader theoretical perspective, the observed moderation effects should not be interpreted as evidence for a unidirectional causal pathway between self-concept clarity and intolerance of uncertainty. Given the cross-sectional design and the relatively stable, personality-related nature of both constructs, the findings are more consistent with a reciprocal or interdependent relationship in their association with positive and disorganized schizotypy. Lower self-concept clarity may heighten vulnerability to uncertainty-related distress, thereby amplifying schizotypal features, while elevated intolerance of uncertainty may undermine the maintenance of a coherent self-concept, indirectly contributing to schizotypal expression. Accordingly, the joint presence of low self-concept clarity and elevated intolerance of uncertainty may represent a particularly relevant risk configuration for positive and disorganized schizotypal traits.

In light of recent evidence demonstrating direct associations between intolerance of uncertainty and schizotypal personality traits [35,40], it is also important to consider the possibility that intolerance of uncertainty may function as a mediating mechanism within its framework. Although the present study focused on moderation effects, the observed interaction patterns do not preclude models in which self-concept clarity influences schizotypal traits indirectly via intolerance of uncertainty, or vice versa. Future longitudinal and experimental studies are needed to disentangle these pathways and to test more complex models in which intolerance of uncertainty operates both as a mediator and a moderator.

Finally, although prospective and inhibitory intolerance of uncertainty are conceptually distinct, both facets demonstrated comparable moderation effects in the present study. This convergence may point to a higher-order uncertainty-related vulnerability, whereby anticipatory cognitive engagement with uncertainty and behavioral inhibition in uncertain contexts co-occur and jointly shape the influence of self-concept clarity on schizotypal features. Importantly, this pattern was observed for both positive and disorganized schizotypy, with no differential effects emerging between the two intolerance of uncertainty facets. Given the integrative nature of disorganized schizotypy and the prominence of perceptual and belief-related processes in positive schizotypy, both dimensions may be particularly sensitive to elevated levels of uncertainty intolerance, even in the absence of facet-specific effects.

### Strengths and limitations

The present study offers several novel contributions to the understanding of schizotypal traits and their cognitive-affective underpinnings. First, it is among the first to examine whether intolerance of uncertainty moderates the relationship between self-concept clarity and positive schizotypy—an interaction that has not yet been explored in prior research. While both intolerance of uncertainty and self-concept clarity have been individually associated with psychopathology, particularly within anxiety and psychosis-spectrum disorders, their combined effect on schizotypal traits remains poorly understood. Second, by focusing on a non-clinical sample, this study contributes to the growing body of research supporting a dimensional view of psychosis and highlights how subclinical traits can be shaped by the interplay between identity-related and uncertainty-related cognitive processes. Finally, by integrating constructs typically studied in separate fields—such as self-concept clarity in identity development and intolerance of uncertainty in anxiety research—this study offers a more comprehensive framework for understanding vulnerability mechanisms that may precede the onset of clinical symptoms.

Although Facebook-based recruitment enabled access to a heterogeneous non-clinical sample, the overrepresentation of women indicates that the sample was not fully representative, which may limit the generalizability of the findings.

In addition, the present study did not assess or control for broader negative affective traits or symptoms, such as neuroticism, general distress, anxiety, and depression, which may represent important confounding variables. Previous research indicates that both anxiety and depressive symptoms are associated with lower self-concept clarity as well as with higher levels of intolerance of uncertainty [41,42]. Moreover, recent evidence suggests that intolerance of uncertainty is associated not only with paranoia and psychosis-spectrum symptoms, but also with anxiety and depressive symptoms across clinical and non-clinical samples [43,44]. Consequently, we cannot determine whether the observed associations are specific to intolerance of uncertainty and self-concept clarity, or whether they partly reflect broader negative affectivity. Future studies would benefit from including measures of neuroticism, anxiety, depression, and general distress to clarify the incremental and specific contribution of intolerance of uncertainty and self-concept clarity to schizotypal traits.

Furthermore, while we conceptualized schizotypy as an analog for schizophrenia, our study did not include individuals with a clinical diagnosis. As a result, our findings cannot be directly generalized to clinical populations. However, a substantial body of research supports the notion that schizotypy reflects a continuum of liability for schizophrenia, both in terms of phenomenology and underlying neurocognitive mechanisms [2,45]. From this perspective, studying schizotypal traits in non-clinical populations offers a valuable window into the early and subclinical processes that may contribute to the development of psychotic disorders. We therefore believe that our findings provide a meaningful foundation for future research on schizophrenia and related conditions.

### Conclusions and future directions

We believe our findings have practical implications. Future research should explore these associations longitudinally to establish causal pathways and develop interventions aimed at enhancing self-concept clarity to mitigate schizotypal symptoms. Given that intolerance of uncertainty emerged as a moderating factor, targeted interventions could focus on reducing intolerance of uncertainty and fostering resilience in individuals at risk for schizophrenia. Improving one’s ability to manage anxiety related to uncertainty may, in turn, facilitate addressing self-related difficulties that contribute to positive schizotypy.

A mediational pathway is also theoretically plausible. For example, low self-concept clarity may contribute to greater intolerance of uncertainty, which may in turn relate to schizotypal traits, or intolerance of uncertainty may undermine the maintenance of a coherent self-concept. However, the cross-sectional design of the present study does not allow strong conclusions about temporal ordering among these constructs. Accordingly, we treated moderation as the primary analytic framework and regard mediational pathways as an important question for future longitudinal and experimental studies.

In addition, future research may benefit from examining the developmental factors that shape self-concept clarity and vulnerability to psychopathology. In particular, prior work has highlighted the role of childhood trauma in the development of self-concept disturbances and later psychopathology [13]. Investigating the interplay between early adverse experiences, self-concept clarity, and intolerance of uncertainty may provide valuable insight into the developmental pathways underlying schizotypal traits and help contextualize the present findings within a broader developmental framework.

In future studies, we aim to extend our research to clinical populations and compare clinical groups with subclinical individuals. Additionally, we plan to investigate the relationship between intolerance of uncertainty, positive schizotypy, and conspiracy beliefs, a highly relevant topic in contemporary psychological research.

## Supporting information

S1 Table S1A Table. Linear regression results for ideas of reference with prospective IU as moderator. **S1B Table.** Linear regression results for ideas of reference with inhibitory IU as moderator.
(PDF)

S2 Table S2A Table. Linear regression results for suspiciousness with prospective IU as moderator. **S2B Table.** Linear regression results for suspiciousness with inhibitory IU as moderator.
(PDF)
