# Supplementary material for: The uncertain self: Intolerance of uncertainty moderates the association of both positive and disorganized schizotypal traits with self-concept clarity in a non-clinical sample
Source: PLoS One. 2026 Jul 2;21(7):e0352650. doi: 10.1371/journal.pone.0352650 (PMC13327182; doi:10.1371/journal.pone.0352650)
Supplement: S1 Table — Linear regression results for ideas of reference with prospective IU as moderator. S1B Table. Linear regression results for ideas of reference with inhibitory IU as moderator. (PDF) [file pone.0352650.s001.pdf]

S1A Table. **Linear regression results for ideas of reference with prospective IU as moderator**

**Model**	**R**	**R2**	**Adj. R2**	**RMSE**
M0	0.000	0.000	0.000	4.136
M1	0.627	0.393	0.379	3.258

	**Sum of Squares**	**df**	**Mean Square**	**F**	**p**
Regression	2,111	7	301.57	28.41	<.001
Residual	3,259	307	10.62		
Total	5,370	314			

**Predictor**	**B**	**SE B**	**ß**	**t**	**p**
Intercept	8.809	5.172		1.703	0.090
SCCS	-0.166	0.102	-0.364	-1.636	0.103
IUS PRO	-0.174	0.210	-0.197	-0.828	0.408
Negative	0.137	0.030	0.269	4.584	<.001
Disorganized	0.209	0.039	0.310	5.319	<.001
Age	0.024	0.016	0.070	1.542	0.124
Sex	0.477	0.447	0.048	1.067	0.287
SCCS x IUS PRO	0.006	0.005	0.350	1.215	0.225

Note. N = 315. SCCS = Self-Concept Clarity. IUS PRO = Prospective Intolerance of Uncertainty. Negative = Negative dimension of schizotypy. Disorganized = Disorganized dimension of schizotypy. B = unstandardized coefficients; SE B = standard error of B; ß = standardized coefficient.

S1B Table. **Linear regression results for ideas of reference with inhibitory IU as moderator**

Model	R	R2	Adj. R2	RMSE
M0	0.000	0.000	0.000	4.136
M1	0.646	0.417	0.404	3.194

	Sum of Squares	df	Mean Square	F	p
Regression	2,239	7	319.82	31.35	<.001
Residual	3,131	307	10.20		
Total	5,370	314			

Predictor	B	SE B	ß	t	p
Intercept	5.159	4.507		1.145	0.253
SCCS	-0.114	0.086	-0.248	-1.317	0.189
IUS INH	-0.040	0.260	-0.034	-0.155	0.877
Negative	0.117	0.029	0.229	4.024	<.001
Disorganized	0.212	0.039	0.314	5.453	<.001
Age	0.021	0.015	0.060	1.341	0.181
Sex	0.530	0.438	0.053	1.208	0.228
SCCS x IUS INH	0.006	0.006	0.244	1.129	0.260

Note. N = 315. SCCS = Self-Concept Clarity. IUS INH = Inhibitory Intolerance of Uncertainty. Negative = Negative dimension of schizotypy. Disorganized = Disorganized dimension of schizotypy. B = unstandardized coefficients; SE B = standard error of B; ß = standardized coefficient.
