# Supplementary material for: The uncertain self: Intolerance of uncertainty moderates the association of both positive and disorganized schizotypal traits with self-concept clarity in a non-clinical sample
Source: PLoS One. 2026 Jul 2;21(7):e0352650. doi: 10.1371/journal.pone.0352650 (PMC13327182; doi:10.1371/journal.pone.0352650)
Supplement: S2 Table — Linear regression results for suspiciousness with prospective IU as moderator. S2B Table. Linear regression results for suspiciousness with inhibitory IU as moderator. (PDF) [file pone.0352650.s002.pdf]

S2A Table. **Linear regression results for suspiciousness with prospective IU as moderator**

**Model**	**R**	**R2**	**Adj. R2**	**RMSE**
M0	0.000	0.000	0.000	2.728
M1	0.624	0.390	0.376	2.156

	**Sum of Squares**	**df**	**Mean Square**	**F**	**p**
Regression	910.5	7	130.069	27.99	<.001
Residual	1,426.5	307	4.647		
Total	2,337.0	314			

**Predictor**	**B**	**SE B**	**ß**	**t**	**p**
Intercept	5.069	3.422		1.481	0.140
SCCS	-0.125	0.067	-0.413	-1.853	0.065
IUS PRO	-0.125	0.139	-0.214	-0.897	0.371
Negative	0.102	0.020	0.303	5.143	<.001
Disorganized	0.104	0.026	0.233	3.983	<.001
Age	0.015	0.010	0.065	1.434	0.153
Sex	0.292	0.296	0.044	0.987	0.324
SCCS x IUS PRO	0.005	0.003	0.523	2.196	0.072

Note. N = 315. SCCS = Self-Concept Clarity. IUS PRO = Prospective Intolerance of Uncertainty. Negative = Negative dimension of schizotypy. Disorganized = Disorganized dimension of schizotypy. B = unstandardized coefficients; SE B = standard error of B; ß = standardized coefficient.

S2B Table. **Linear regression results for suspiciousness with inhibitory IU as moderator**

Model	R	R2	Adj. R2	RMSE
M0	0.000	0.000	0.000	2.728
M1	0.637	0.406	0.392	2.127

	Sum of Squares	df	Mean Square	F	p
Regression	948.5	7	135.506	29.96	<.001
Residual	1,388.5	307	4.523		
Total	2,337.0	314			

Predictor	B	SE B	ß	t	p
Intercept	1.371	3.001		0.457	0.648
SCCS	-0.053	0.057	-0.176	-0.925	0.356
IUS INH	-0.005	0.173	-0.006	-0.028	0.977
Negative	0.099	0.019	0.293	5.104	<.001
Disorganized	0.112	0.026	0.251	4.315	<.001
Age	0.013	0.010	0.056	1.242	0.215
Sex	0.319	0.292	0.049	1.092	0.276
SCCS x IUS INH	0.005	0.004	0.285	1.306	0.193

Note. N = 315. SCCS = Self-Concept Clarity. IUS INH = Inhibitory Intolerance of Uncertainty. Negative = Negative dimension of schizotypy. Disorganized = Disorganized dimension of schizotypy. B = unstandardized coefficients; SE B = standard error of B; ß = standardized coefficient.
