# Supplementary material for: The uncertain self: Intolerance of uncertainty moderates the association of both positive and disorganized schizotypal traits with self-concept clarity in a non-clinical sample
Source: PLoS One. 2026 Jul 2;21(7):e0352650. doi: 10.1371/journal.pone.0352650 (PMC13327182; doi:10.1371/journal.pone.0352650)
Supplement: Attachment — Submitted filename: Response to Reviewers.pdf [file pone.0352650.s004.pdf]

Additional Editor Comments:

Both reviewers referred to a systematic review by Morriss et al, 2025, particular note should be made of these findings to incorporate into the manuscript. In addition, all of the points raised by the reviewers should be addressed

[Note: HTML markup is below. Please do not edit.]

Reviewers' comments:

Reviewer's Responses to Questions

Comments to the Author

1. Is the manuscript technically sound, and do the data support the conclusions?

The manuscript must describe a technically sound piece of scientific research with data that supports the conclusions. Experiments must have been conducted rigorously, with appropriate controls, replication, and sample sizes. The conclusions must be drawn appropriately based on the data presented.

Reviewer #1: Partly

Reviewer #2: Partly

---

2. Has the statistical analysis been performed appropriately and rigorously?

Reviewer #1: Yes

Reviewer #2: Yes

---

3. Have the authors made all data underlying the findings in their manuscript fully available?

The PLOS Data policy requires authors to make all data underlying the findings described in their manuscript fully available without restriction, with rare exception (please refer to the Data Availability Statement in the manuscript PDF file). The data should be provided as part of the manuscript or its supporting information, or deposited to a public repository. For example, in addition to summary statistics, the data points behind means, medians and variance measures should be available. If there are restrictions on publicly sharing data—e.g. participant privacy or use of data from a third party—those must be specified.

Reviewer #1: Yes

Reviewer #2: Yes

4. Is the manuscript presented in an intelligible fashion and written in standard English?

PLOS ONE does not copyedit accepted manuscripts, so the language in submitted articles must be clear, correct, and unambiguous. Any typographical or grammatical errors should be corrected at revision, so please note any specific errors here.

Reviewer #1: Yes

Reviewer #2: Yes

---

5. Review Comments to the Author

Please use the space provided to explain your answers to the questions above. You may also include additional comments for the author, including concerns about dual publication, research ethics, or publication ethics. (Please upload your review as an attachment if it exceeds 20,000 characters)

All the alterations are highlighted in green in the manuscript.

Reviewer #1:

This manuscript "The uncertain self: intolerance of uncertainty moderates the association of positive schizotypal traits and self-concept clarity" reports moderation role of intolerance of uncertainty (IU) in relationship between self-concept clarity (SCC) and positive schizotypy in a sample of 315 normal adults. The manuscript is well written however it has **concerns related to its objectives and methodology**.

R1.1

The authors' decision to limit the study to the association between positive schizotypal personality and SCC needs further theoretical justification. Given that SCC and IU are reported to be related to all three schizotypal dimensions in the literature, as well as in the authors' own correlation data, the current justification—which primarily links IU to positive symptoms—does not fully explain the exclusion of the negative and disorganized dimensions from the primary analysis. Exploring the moderating role of IU across all three schizotypal dimensions would significantly enhance the theoretical breadth and informativeness of the manuscript.

Thank you for this insightful comment. We agree that our original rationale for focusing exclusively on the positive dimension of schizotypy required stronger theoretical justification. Our initial emphasis on the positive dimension was based on its more direct conceptual association with intolerance of uncertainty. However, we appreciate the reviewer's point that

both self-concept clarity and intolerance of uncertainty have been linked to all three schizotypal dimensions, both in the broader literature and in our own correlational findings.

In response to this valuable suggestion, we have expanded the introduction and the moderation analyses to include the negative and disorganized dimensions as well (both as covariates and outcomes). We believe this addition not only strengthens the theoretical scope of the manuscript but also provides a more comprehensive understanding of schizotypy and the distinctions among its dimensions. The results of these additional analyses have now been incorporated into the revised manuscript and are presented in the Introduction (pp. 2) and Results (pp. 10) sections, along with the new tables.

> Like schizophrenia, schizotypy can be divided into three dimensions based on factor analysis: positive, negative, and disorganized schizotypy [4]. Positive schizotypy involves an excess of phenomena that should not be present, such as unusual experiences, magical thinking, or ideas of reference [5]. In contrast, negative schizotypy reflects deficits in expected functions, such as avolition or an absence of close relationships [6]. Disorganized schizotypy pertains to disturbances in the regulation of thought and behavior [7]. Notably, higher levels of disorganization may predict increased positive and negative traits, and vice versa [6]. As a result, disorganization must be considered to fully differentiate the dimensions of schizotypy.

First, extending the moderation model with the negative and disorganized dimensions did not change the conclusions regarding the moderating role of intolerance of uncertainty in the relationship between self-concept clarity and positive schizotypy. Second, intolerance of uncertainty moderated the negative association of self-concept clarity with disorganized schizotypy too: the association was most pronounced at low levels of intolerance of uncertainty, while it was weaker or absent at medium or high levels of intolerance of uncertainty, respectively. Third, intolerance of uncertainty did not significantly moderate the negative association between self-concept clarity and negative schizotypy. All tables have been edited and expanded to include the two additional dimensions. In addition, we added a novel, single figure summarising moderation analyses for all the schizotypy dimensions, showing both associations in the raw data by tertile splits and the estimated slopes of schizotypy - self-concept clarity as a continuous function of intolerance of uncertainty.

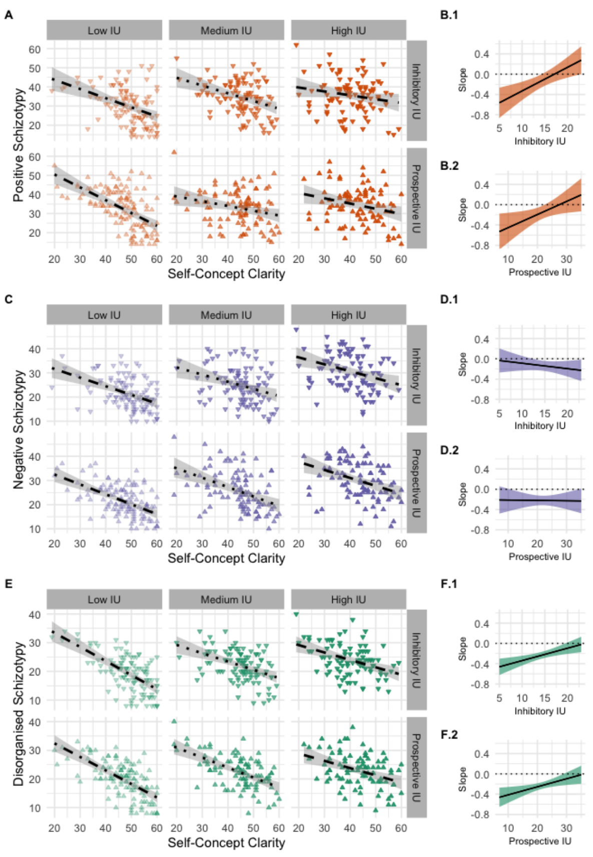

Figure 1. Association of self-concept clarity with schizotypy dimensions as a function of intolerance of uncertainty (IU). The ribbons indicate 95% confidence intervals. **A:** the association between self-concept clarity and positive schizotypy varied across low, medium, and high IU subgroups. **B:** the estimated slope coefficient between self-concept clarity and positive schizotypy as a function of IU. **C:** association between self-concept clarity and negative schizotypy did not significantly vary across low, medium, and high IU subgroups. **D:** estimated slope coefficient between self-concept clarity and negative schizotypy as a function of IU. **E:** the association between self-concept clarity and disorganised schizotypy varied across low, medium, and high IU subgroups. **F:** estimated slope coefficient between self-concept clarity and disorganised schizotypy as a function of IU. Low, medium, and high levels of Intolerance of Uncertainty were based on tertile splits for visualisation (panels A, C, E). Slope estimates were extracted from a linear model where a dimension of schizotypy was regressed on self-concept clarity, intolerance of uncertainty, and their interaction; the models were adjusted for age, sex, and the other dimensions of schizotypy (panels B, D, F).

A summary of the results extracted from the Discussion (pp. 21) is shown below:

> The present study provides novel insights into the interplay between self-concept clarity, intolerance of uncertainty, and schizotypal traits. Consistent with prior research, self-concept clarity was negatively associated with all three dimensions of schizotypy, indicating that individuals with a less coherent and stable self-concept tend to report elevated positive, negative, and disorganized schizotypal features. These findings align with theoretical and empirical work emphasizing self-disturbances as a

core characteristic of schizotypy and related psychopathological conditions [12,14-16], and further support the relevance of self-concept clarity as an important factor in understanding schizotypal personality traits.

R1.2

The inclusion of only the disorganized dimension as a covariate in the regression equation predicting positive schizotypy lacks clear justification. For methodological consistency, the authors should consider either including both negative and disorganized dimensions as covariates or, alternatively, omitting both from the primary moderation analysis. Clarification on the rationale for the current choice is necessary.

Thank you for this important comment. We agree that, for methodological consistency, the regression model should either control for both of the remaining schizotypy dimensions or for neither of them. In response to the reviewer's suggestion, we chose to include both the negative and disorganized dimensions as covariates in the revised analyses.

This decision was guided by both empirical and theoretical considerations. First, the three schizotypy dimensions are substantially interrelated in our sample and therefore cannot be treated as entirely independent constructs (see Table 2 for a correlation matrix). Controlling for the remaining dimensions therefore allows us to more clearly examine whether the observed effects are specific to positive schizotypy rather than reflecting variance shared across schizotypy dimensions. In addition, prior work has highlighted the particular importance of the disorganized dimension within the broader schizotypy structure (e.g., Meehl, 1989; Polner et al., 2021). Taken together, these considerations supported our decision to include both non-target dimensions as covariates.

We have included the alterations in the revised manuscript (pp. 2; 12).

> Like schizophrenia, schizotypy can be divided into three dimensions based on factor analysis: positive, negative, and disorganized schizotypy [4]. Positive schizotypy involves an excess of phenomena that should not be present, such as unusual experiences, magical thinking, or ideas of reference [5]. In contrast, negative schizotypy reflects deficits in expected functions, such as avolition or an absence of close relationships [6]. Disorganized schizotypy pertains to disturbances in the regulation of thought and behavior [7]. Notably, higher levels of disorganization may predict increased positive and negative traits, and vice versa [6]. As a result, disorganization must be considered to fully differentiate the dimensions of schizotypy.
>
> [...] Age and sex were entered as control variables. Additionally, when a given schizotypy dimension was treated as the dependent variable, the remaining two schizotypy dimensions were controlled for (e.g., in models predicting positive schizotypy, negative and disorganized schizotypy were included as covariates).

R1.3

The interpretation of the moderation effect, particularly the assumption of IU as an antecedent influencing the relationship between SCC and schizotypal personality requires stronger justification. Since both IU and SCC are stable personality-related constructs, establishing a clear antecedent-consequent relationship is difficult in a cross-sectional study. To support the current interpretation, the authors should cite longitudinal or experimental evidence showing IU as an antecedent of SCC. Failing this, the authors are encouraged to interpret the interactional relationship in both directions.

Thank you for this thoughtful comment. We agree that the interpretation of the moderation effect requires greater caution and that our cross-sectional design does not permit conclusions about a unidirectional causal or antecedent–consequent relationship between intolerance of uncertainty and self-concept clarity. As the reviewer rightly notes, both constructs are relatively stable and personality-related, which makes it difficult to establish temporal precedence on the basis of the present data alone.

In response to this concern, we have revised the Discussion (pp. 22) to avoid implying a causal interpretation and to acknowledge the possibility of a reciprocal or bidirectional relationship between these constructs. Specifically, we now state:

> From a broader theoretical perspective, the observed moderation effects should not be interpreted as evidence for a unidirectional causal pathway between self-concept clarity and intolerance of uncertainty. Given the cross-sectional design and the relatively stable, personality-related nature of both constructs, the findings are more consistent with a reciprocal or interdependent relationship in their association with positive and disorganized schizotypy. Lower self-concept clarity may heighten vulnerability to uncertainty-related distress, thereby amplifying schizotypal features, while elevated intolerance of uncertainty may undermine the maintenance of a coherent self-concept, indirectly contributing to schizotypal expression. Accordingly, the joint presence of low self-concept clarity and elevated intolerance of uncertainty may represent a particularly relevant risk configuration for positive and disorganized schizotypal traits.

R1.4

Given that the current results, alongside recent literature (e.g., Kumar et al., 2025; Morriss et al., 2025), demonstrate a direct relationship between IU and schizotypal personality, the authors should consider exploring a more complex model. A moderated mediation analysis might offer a richer understanding of the combined influence of IU. At minimum, the discussion of the moderation effects should also briefly address the potential for a mediating role of IU, which could lead to a more comprehensive theoretical explanation.

Reference

Kumar et al., 2025. The Role of Intolerance of Uncertainty and Religiousness in Schizotypal Personality and Life Satisfaction: A Cross-Sectional Study. The Journal of psychology,

159(2), 92–110.
Morriss et al. (2025). Intolerance of uncertainty and psychosis: A systematic review. The British journal of clinical psychology, 64(2), 344–354.

Thank you for this valuable suggestion. We appreciate the reviewer's recommendation to consider a more complex model in light of the growing literature demonstrating direct associations between intolerance of uncertainty and schizotypal personality traits. We have carefully reviewed the cited studies and agree that moderated mediation models may provide an important direction for future research.

At the same time, our primary aim in the present study was to test a theoretically focused moderation model concerning the joint role of self-concept clarity and intolerance of uncertainty in relation to schizotypal traits. We were cautious about extending the analyses to a more complex conditional process model within the current manuscript, as the cross-sectional design does not allow for strong inferences regarding indirect pathways or temporal ordering among the variables. We therefore felt that introducing a moderated mediation analysis at this stage could risk overinterpreting the data beyond what the design can adequately support.

In response to the reviewer's suggestion, we have expanded the Discussion (pp. 23) to acknowledge the potential mediating role of intolerance of uncertainty and to situate our findings within this broader theoretical framework. We now state:

> In light of recent evidence demonstrating direct associations between intolerance of uncertainty and schizotypal personality traits [44,45], it is also important to consider the possibility that intolerance of uncertainty may function as a mediating mechanism within its framework. Although the present study focused on moderation effects, the observed interaction patterns do not preclude models in which self-concept clarity influences schizotypal traits indirectly via intolerance of uncertainty, or vice versa. Future longitudinal and experimental studies are needed to disentangle these pathways and to test more complex models in which intolerance of uncertainty operates both as a mediator and a moderator.

Reviewer #2:

This study makes a valuable contribution to the literature by addressing the association between self-concept clarity, positive schizotypy and the effect of intolerance of uncertainty. The authors recruited a large non-clinical sample and assessed schizotypal traits (SPQ-BR), self-concept clarity (SCCS), and intolerance of uncertainty (IUS). Their correlational analyses indicated that lower self-concept clarity was negatively associated with higher levels across all schizotypy dimensions, as well as with both intolerance of uncertainty subscales. The subsequent moderation analyses further demonstrated that higher levels of intolerance of uncertainty attenuated the negative relationship between self-concept clarity and positive schizotypy. Findings suggest that intolerance of uncertainty may buffer, the extent to which reduced self-concept clarity relates to positive schizotypal traits.

R2.1

Could the authors clarify whether they controlled for anxiety, or depressive symptoms in their study? Prior literature suggests that these factors are associated with both intolerance of uncertainty and self-concept clarity, and accounting for them could strengthen the interpretation of the findings.

Thank you for raising this important point. We did not include measures of anxiety or depressive symptoms in the present study. As mentioned in the Procedure section, we analyzed data from a study primarily designed to estimate the associations between schizotypal traits and memory performance, which will be reported in a separate paper. Because the original study already included three questionnaires and a behavioral test to assess the variables of interest, we decided not to add further measures, as we were concerned about participant burden potentially leading to a higher dropout rate.

We agree that these variables may be relevant, given that prior research has linked them to both intolerance of uncertainty and self-concept clarity, and that accounting for them could further refine the interpretation of the findings.

In response to the reviewer's comment, we have acknowledged this issue in the Strengths and Limitations section (pp. 23) of the revised manuscript as a limitation of the present study and a direction for future research:

> In addition, the present study did not assess or control for symptoms of anxiety and depression, which may represent important confounding variables. Previous research indicates that both anxiety and depressive symptoms are associated with lower self-concept clarity as well as with higher levels of intolerance of uncertainty [45,46]. Consequently, it cannot be ruled out that the observed associations were partly influenced by these broader affective factors. Future studies would benefit from including measures of anxiety and depression to clarify the specificity of the relationships among these variables.

R2.2

Please report the specific inclusion and exclusion criteria used in participant recruitment. In particular, did the authors exclude individuals with current or past psychiatric diagnoses, and if so, how was this assessed? On page 16, the authors state that the study did not include individuals with a clinical diagnosis. Could the authors clarify how this was determined (e.g., screening questions, self-report, exclusion criteria)?

Thank you for this important comment. We agree that the inclusion and exclusion criteria, as well as the procedure used to determine clinical status, should be described more clearly.

In response, we have revised the recruitment section (pp. 6) to provide a more detailed explanation of participant eligibility and screening procedures. Specifically, we now state:

> Participants aged 18 and over were recruited between September 4 and December 31, 2022, via announcements posted on the research team's Facebook page.

The study targeted a non-clinical community sample, and any adult was eligible to participate. Individuals under the age of 18 or those who self-reported a current or past psychiatric diagnosis were excluded; an automated screening mechanism prevented ineligible respondents from completing the questionnaire.

R2.3

It would be helpful if the manuscript included the mean and standard deviation for each schizotypy dimension, the SCCS, and the IUS subscales.

Thank you for this suggestion. In response, we have added a table (Table 1 on page 10) to the Results section reporting the means and standard deviations for each schizotypy dimension, as well as for the Self-Concept Clarity Scale and the Intolerance of Uncertainty Scale subscales. This information has been included to provide a clearer descriptive overview of the study variables.

The means and standard deviations of the used scales can be found in Table 1.

**Descriptive**	**SPQ POS**	**SPQ NEG**	**SPQ DIS**	**SCCS**	**IUS PRO**	**IUS INH**
Mean	33.21	25.33	21.61	44.87	21.12	13.70
St. Deviation	9.675	8.097	6.127	9.031	4.689	3.526

Note. N = 315. SPQ POS = Schizotypy Personality Questionnaire Positive Dimension. SPQ NEG = Schizotypy Personality Questionnaire Negative Dimension. SPQ DIS = Schizotypy Personality Questionnaire Disorganized Dimension. SCCS = Self-Concept Clarity. IUS PROS = Prospective intolerance of uncertainty. IUS INH = Inhibitory intolerance of uncertainty.

R2.4

A recent systematic review (Morriss et al., 2025) demonstrated stronger evidence linking intolerance of uncertainty to paranoia-related symptoms in nonclinical samples. In light of this, the authors may wish to conduct supplementary analyses focusing on SPQ subscales that most closely reflect paranoid ideation (e.g., suspiciousness, ideas of reference). This could offer additional insights into the specificity of the observed effects.

Morriss, J., Butler, D., & Ellett, L. (2025). Intolerance of uncertainty and psychosis: A systematic review. The British journal of clinical psychology, 64(2), 344–354. https://doi.org/10.1111/bjc.12509

Thank you for this valuable suggestion. We agree that, in light of recent evidence indicating a particularly strong link between intolerance of uncertainty and paranoia-related symptoms in nonclinical samples, supplementary analyses focusing on SPQ subscales related to paranoid ideation could provide additional insight into the specificity of the observed effects.

In response, we conducted additional regression analyses examining the subscales most closely related to paranoid ideation, namely ideas of reference and suspiciousness. Given the broader scope and added complexity of these analyses relative to the main aims of the manuscript, we chose to report them in a separate Supplementary Analyses section (pp. 20), with the corresponding tables presented in the Supplementary Materials.

> In light of a recent systematic review linking intolerance of uncertainty to paranoia-related symptoms in non-clinical samples [39], we conducted supplementary linear regression analyses to clarify the associations between the relevant subscales of positive schizotypy (ideas of reference and suspiciousness) and the other variables under study. In these models, the two subscales were examined separately as dependent variables, resulting in four regression models examining ideas of reference and suspiciousness in relation to the prospective and inhibitory dimensions of intolerance of uncertainty. As in the main analyses, age, sex, and the negative and disorganized schizotypy dimensions were included as covariates.
>
> Across the supplementary models, similar patterns emerged for the two schizotypy subscales examined. Both ideas of reference and suspiciousness were primarily associated with the other schizotypy dimensions: the negative and disorganized dimensions showed significant positive associations, whereas self-concept clarity, prospective and inhibitory intolerance of uncertainty, and their interaction were not significant predictors in either model. Detailed results of these supplementary regression analyses are reported in the Supplementary Materials.

R2.5

The correlation analyses indicate significant associations between negative schizotypy, self-concept clarity, and intolerance of uncertainty. Could the authors elaborate on the rationale for not including negative schizotypy as a covariate in the moderation models?

Thank you for this important observation. In the original analyses, we included only the disorganized dimension as a covariate based on prior work suggesting that it may occupy a more central position within the schizotypy network structure (e.g., Polner et al., 2021). However, we agree that, given the significant associations observed for negative schizotypy, this choice required stronger justification.

In response to the comments raised by both reviewers, we revised the moderation models to include both negative and disorganized schizotypy as covariates. As shown in our correlation matrix, the three schizotypy dimensions are substantially interrelated and therefore cannot be considered fully independent. Controlling for the non-target dimensions allows us to test more clearly whether the observed effects are specific to positive schizotypy, rather than reflecting variance shared across schizotypy dimensions.

First, extending the moderation model with the negative and disorganized dimensions did not change the conclusions regarding the moderating role of intolerance of uncertainty in the relationship between self-concept clarity and positive schizotypy. Second, intolerance of uncertainty moderated the negative association of self-concept clarity with disorganized schizotypy too: the association was most pronounced at low levels of intolerance of uncertainty, while it was weaker or absent at medium or high levels of intolerance of uncertainty, respectively. Third, intolerance of uncertainty did not significantly moderate the negative association between self-concept clarity and negative schizotypy. All tables have been edited and expanded to include the two additional dimensions. In addition, we added a novel, single figure summarising moderation analyses for all the schizotypy dimensions, showing both associations in the raw data by tertile splits and the estimated slopes of schizotypy - self-concept clarity as a continuous function of intolerance of uncertainty.

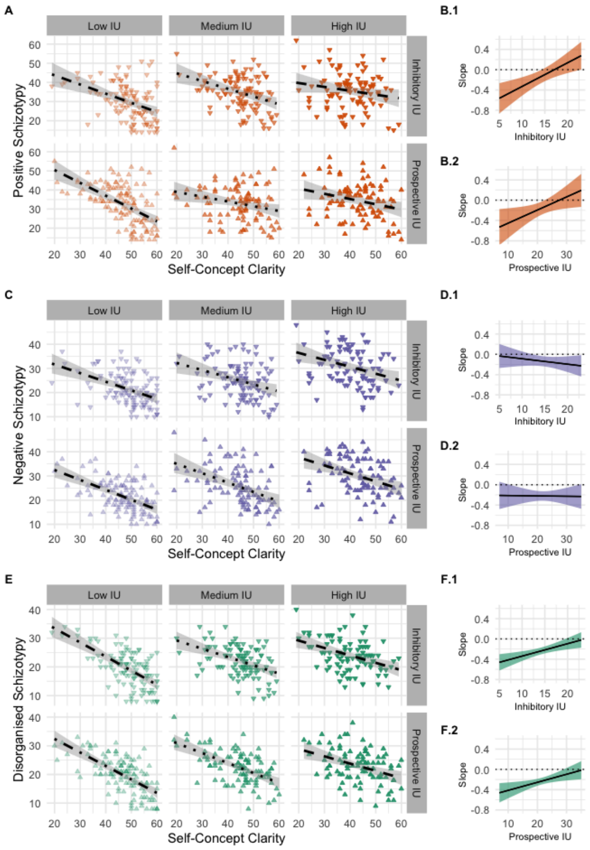

Figure 1. Association of self-concept clarity with schizotypy dimensions as a function of intolerance of uncertainty (IU). The ribbons indicate 95% confidence intervals. **A:** the association between self-concept clarity and positive schizotypy varied across low, medium, and high IU subgroups. **B:** the estimated slope coefficient between self-concept clarity and positive schizotypy as a function of IU. **C:** association between self-concept clarity and negative schizotypy did not significantly vary across low, medium, and high IU subgroups. **D:** estimated slope coefficient between self-concept clarity and negative schizotypy as a function of IU. **E:** the association between self-concept clarity and disorganised schizotypy varied across low, medium, and high IU subgroups. **F:** estimated slope coefficient between self-concept clarity and disorganised schizotypy as a function of IU. Low, medium, and high

levels of Intolerance of Uncertainty were based on tertile splits for visualisation (panels A, C, E). Slope estimates were extracted from a linear model where a dimension of schizotypy was regressed on self-concept clarity, intolerance of uncertainty, and their interaction; the models were adjusted for age, sex, and the other dimensions of schizotypy (panels B, D, F).

R2.6

On page 9, the authors state that "all associations were strongly negative." However, the reported Spearman's rho values (−0.386, −0.482, −0.528) fall within the moderate range. Please consider revising this description to more accurately reflect the magnitude of the correlations.

Thank you for pointing this out. We agree that describing these associations as "strongly negative" was not accurate given the reported effect sizes. We have revised the relevant section of the manuscript to describe the correlations more appropriately in line with their magnitude.

R2.7

In Table 1, the correlation between prospective IUS and positive schizotypy is non-significant (p = .229). Could the authors provide an explanation or interpretation for this finding

Thank you for this thoughtful question. We agree that this non-significant association warrants brief interpretation. While a non-significant result cannot be conclusively explained within a frequentist framework, it may reflect meaningful differences between the two facets of intolerance of uncertainty and their relationship to schizotypal traits.

In response, we have added the following interpretive note to the manuscript:

> At bivariate level, the absence of a significant association between prospective intolerance of uncertainty and positive schizotypy may reflect the distinct psychological processes underlying these constructs. Prospective intolerance of uncertainty captures an active, future-oriented cognitive engagement with uncertainty, whereas positive schizotypy predominantly involves perceptual anomalies and unusual belief systems that may not rely on anticipatory uncertainty processing. Importantly, the lack of a zero-order correlation does not preclude conditional effects, as moderation can occur independently of direct associations between variables.

R2.8

Since the authors performed separate moderation analyses for the inhibitory and prospective IUS subscales, it would be beneficial to expand the Discussion section to address possible underpinnings of these two facets of intolerance of uncertainty

Thank you for this valuable suggestion. We agree that, because the moderation analyses were conducted separately for the inhibitory and prospective dimensions of intolerance of

uncertainty, the Discussion should more clearly address the potential conceptual differences between these two facets and their distinct roles in relation to schizotypal traits.

In response, we have expanded the Discussion to elaborate on the possible psychological underpinnings of inhibitory and prospective intolerance of uncertainty (please see our response to your previous point R2.7 right above). In particular, we discuss that prospective intolerance of uncertainty reflects a more active, future-oriented cognitive engagement with uncertainty, whereas inhibitory intolerance of uncertainty is more closely related to behavioral paralysis, avoidance, and difficulty acting under uncertain conditions. We believe that acknowledging these distinctions helps provide a more nuanced interpretation of the differential patterns observed in our analyses.

R2.9

In the abstract, the authors state that inhibitory intolerance of uncertainty significantly moderated the relationship between self-concept clarity and positive schizotypy. However, according to the reported results, the prospective IU subscale also yielded a significant moderation effect. Please revise the abstract to reflect both findings and provide a balanced interpretation of the two moderation effects.

Thank you for pointing this out. In response, we have revised the abstract to reflect both significant moderation effects and to provide a more balanced summary of the findings. The updated wording now acknowledges that both inhibitory and prospective intolerance of uncertainty significantly moderated the relationship between self-concept clarity and positive schizotypy:

> Our objective was to investigate the relationship between self-concept clarity, intolerance of uncertainty, and schizotypal traits, with a focus on whether intolerance of uncertainty moderates the association between self-concept clarity and positive schizotypy in a non-clinical context. A sample of 315 adults (on average 43 (SD=12) years, 247 women) completed the Self-Concept Clarity Scale, the Intolerance of Uncertainty scale, and the Schizotypal Personality Questionnaire - Brief Revised. Lower self-concept clarity was significantly associated with higher levels of positive, negative, and disorganized schizotypy, as well as with greater inhibitory intolerance of uncertainty. Importantly, a moderation analysis revealed that intolerance of uncertainty significantly altered the strength of the negative relationship between self-concept clarity and both positive and disorganized schizotypy. Specifically, this association was strongest among individuals with low levels of intolerance of uncertainty. In contrast, at higher levels of intolerance of uncertainty, the negative relationship between self-concept clarity and both positive and disorganized schizotypy was weaker. This pattern suggests that, among individuals with lower tolerance for uncertainty, a better-defined self-concept is more strongly associated with lower levels of positive and disorganized schizotypy. These results underscore the importance of considering both self-structure and uncertainty tolerance in models of schizotypy and psychosis risk. Our findings suggest that self-concept clarity and intolerance of uncertainty are interrelated cognitive factors that synergistically explain variance in schizotypal traits. As intolerance of uncertainty is a potentially modifiable cognitive process, the results have implications for early interventions aimed at

reducing psychosis risk by improving self-concept clarity and tolerance for uncertainty. Although the study used a non-clinical sample, it supports a dimensional approach to psychosis and highlights key targets for future interventional research.

R2.10

For future directions, the authors may consider discussing research on the interplay between childhood trauma, self-concept clarity, and psychopathology, as highlighted in prior work (e.g., Evans et al., 2015). This line of inquiry may provide additional context for understanding the developmental pathways underlying schizotypal traits.

Thank you for this valuable suggestion. We agree that considering developmental antecedents, particularly childhood trauma, can provide important context for understanding the emergence of self-concept disturbances and schizotypal traits.

In response, we have incorporated this perspective into the Conclusions and Future Directions section. Specifically, we now state:

> In addition, future research may benefit from examining the developmental factors that shape self-concept clarity and vulnerability to psychopathology. In particular, prior work has highlighted the role of childhood trauma in the development of self-concept disturbances and later psychopathology [16]. Investigating the interplay between early adverse experiences, self-concept clarity, and intolerance of uncertainty may provide valuable insight into the developmental pathways underlying schizotypal traits and help contextualize the present findings within a broader developmental framework.

R2.11

There appears to be a minor typographical error in the references: 'ref 255' should be 'ref 25' page 5. Please correct this."

Thank you, corrected.

---

6. PLOS authors have the option to publish the peer review history of their article (what does this mean?). If published, this will include your full peer review and any attached files.

If you choose "no", your identity will remain anonymous but your review may still be made public.

Do you want your identity to be public for this peer review? For information about this choice, including consent withdrawal, please see our Privacy Policy.

Reviewer #1: No

Reviewer #2: No
