# Supplementary material for: The uncertain self: Intolerance of uncertainty moderates the association of both positive and disorganized schizotypal traits with self-concept clarity in a non-clinical sample
Source: PLoS One. 2026 Jul 2;21(7):e0352650. doi: 10.1371/journal.pone.0352650 (PMC13327182; doi:10.1371/journal.pone.0352650)
Supplement: Attachment — Submitted filename: Response to Reviewers - Revision 2.pdf [file pone.0352650.s005.pdf]

Reviewer #3:

Thank you for inviting me to review this interesting manuscript. I only have a few minor comments.

Thank you for evaluating our manuscript. We also appreciate the positive evaluation and the constructive points. Below, we reply one-by-one to the points, and attach the revised manuscript with changes highlighted in green.

R3.1

Can the authors refrain from using deficit based language throughout e.g.

'negative schizotypy reflects deficits in expected functions' → **negative schizotypy reflects differences in expected patterns of functioning**
'reduced affective reactivity and motivational deficits' → **reduced affective reactivity and lower motivational engagement**
'facilitate addressing self-related deficits' → **facilitate addressing self-related difficulties**
'more vulnerable to the negative effects of failure'
'cognitive vulnerability'

The field is moving away from using such language as deficit and vulnerability. It is also potentially not accurate to use 'deficit' as there is nothing missing per se, but rather just differences in functioning. Regarding 'vulnerability', it is more appropriate to use the word 'risk'.

Dinishak, J. (2022). The deficit view and its critics. Disability Studies Quarterly, 36(4).

We thank the reviewer for raising this important point. We agree that deficit-based terminology may be unnecessarily pathologizing and, in some contexts, conceptually imprecise. Following the reviewer's suggestion and in line with recent discussions on deficit-based language (Dinishak, 2022), we revised the manuscript throughout to use more neutral and precise terminology. Specifically, we replaced the phrases highlighted by the reviewer as indicated above. We also reviewed the manuscript for similar instances of deficit-based language and revised them where appropriate.

R3.2

'continuum between normality and schizophrenia' please don't use the word 'normality' when describing populations. What is normal? Perhaps, reword to 'schizoptpy exists on a conintuum and is more prevalent in schizophrenia.'

We thank the reviewer for pointing this out. We agree that the term "normality" is not appropriate when describing populations, as it may imply an unnecessary normative distinction. We have therefore revised this phrasing to avoid the term "normality" and to describe schizotypy within a dimensional schizophrenia-spectrum framework. The revision better reflects the dimensional conceptualization of schizotypy while avoiding normative language. Specifically, the sentence now reads:

> "Schizotypy is commonly conceptualized as a dimensional continuum of schizophrenia-spectrum characteristics, ranging from subclinical traits in the general population to clinical manifestations associated with schizophrenia." (pp. 2)

R3.3

How do the authors know that their results are specific to IUS or SCCS. Did the authors measure any other broader negative affective traits (e.g. neuroticism) to control for? If not, please add this limitation and cite relevant papers addressing specificity of IU in relation to psychosis.

Morriss, J., & Ellett, L. (2025). Intolerance of uncertainty, paranoia, and prodromal symptoms: Comparisons between a schizophrenia spectrum disorder, anxiety disorder and non-clinical sample. Psychology and Psychotherapy: Theory, Research and Practice, 98(4), 960-973.

Morriss, J., Gaudiano, B. A., So, S. H., Kingston, J., Lincoln, T., Morris, E. M., & Ellett, L. (2024). Associations between intolerance of uncertainty, paranoia, anxiety, and depression: Evidence from an international multisite sample. Mental Health Science, 2(4), e81.

We thank the reviewer for raising this important issue. We did not assess broader negative affective traits or symptoms, such as neuroticism, general distress, anxiety, or depression, and therefore cannot directly test whether the observed associations are specific to intolerance of uncertainty and self-concept clarity. We have now made this limitation explicit in the manuscript and have revised the discussion accordingly to avoid implying specificity beyond what can be supported by the present data. Specifically, we added the following limitation:

> "In addition, the present study did not assess or control for broader negative affective traits or symptoms, such as neuroticism, general distress, anxiety, and depression, which may represent important confounding variables. Previous research indicates that both anxiety and depressive symptoms are associated with lower self-concept clarity as well as with higher levels of intolerance of uncertainty [41,42]. Moreover, recent evidence suggests that intolerance of uncertainty is associated not only with paranoia and psychosis-spectrum symptoms, but also with anxiety and depressive symptoms across clinical and non-clinical samples [43,44]. Consequently, we cannot determine whether the observed associations are specific to

intolerance of uncertainty and self-concept clarity, or whether they partly reflect broader negative affectivity. Future studies would benefit from including measures of neuroticism, anxiety, depression, and general distress to clarify the incremental and specific contribution of intolerance of uncertainty and self-concept clarity to schizotypal traits." (pp. 28)

R3.4

The authors examine IU using the two dimensions. However,
-there is no rationale for using the two dimensions in the introduction/discussion
-the two dimensions are not mentioned in the hypotheses - even if exploratory
-report total IUS in the questionnaire section and its cronbach alpha please
-what happens when IUS total score is used in the models instead. Please report this in the correlation table as well for future meta-analyses. Given prior, research on IU and psychosis, I think the using the total score is warranted.

Hong, R. Y., & Lee, S. S. (2015). Further clarifying prospective and inhibitory intolerance of uncertainty: Factorial and construct validity of test scores from the Intolerance of Uncertainty Scale. Psychological assessment, 27(2), 605.

We thank the reviewer for this helpful comment. We agree that the rationale for examining the two IU dimensions required clearer theoretical justification. We have revised the Introduction to explain the distinction between prospective IU, reflecting the desire for predictability, and inhibitory IU, reflecting uncertainty-related paralysis or behavioral inhibition. We also added Hong and Lee's findings supporting the two-factor structure of IU and the differential construct validity of the two subscales. We reasoned that this evidence motivates using the two dimensions. Therefore, we revised the hypotheses to explicitly state that prospective and inhibitory IU are examined separately, while noting that their differential moderating effects are exploratory due to the limited prior literature on self-concept clarity, IU dimensions, and schizotypy.

That said, for the sake of completeness, we repeated the analyses using the total score, as requested. We do agree that some readers and meta-analysts might find this information valuable, and analyses with the total IUS score can facilitate comparison with prior literature. We have extended the correlation and regression tables with the results concerning the total IUS score. Overall, the pattern of results were unchanged and these analyses point towards the same conclusions.

R3.5

'This anxiety can contribute to intolerance of uncertainty, which refers to how individuals perceive and react to ambiguous or unpredictable situations'. This section on IU could be expanded to included modern reviews and special issues on IU in relation to mechanisms:

Carleton, R. N. (2016). Fear of the unknown: One fear to rule them all?. Journal of anxiety disorders, 41, 5-21.

Morriss, J., Abend, R., Zika, O., Bradford, D. E., & Mertens, G. (2023). Neural and psychophysiological markers of intolerance of uncertainty. International Journal of Psychophysiology, 184, 94-99.

We thank the reviewer for this helpful suggestion. We have expanded the section introducing intolerance of uncertainty by incorporating contemporary theoretical and mechanistic accounts. Specifically, we now refer to Carleton's conceptualization of fear of the unknown as a transdiagnostic mechanism and to recent work by Morriss and colleagues highlighting neural and psychophysiological markers of intolerance of uncertainty. This revision clarifies why IU may be relevant not only as a cognitive tendency, but also as a broader mechanism involving affective and physiological responses to ambiguity and unpredictability.

> "Another construct that may be relevant to self-related difficulties and schizotypal traits is intolerance of uncertainty. Intolerance of uncertainty refers to a dispositional tendency to perceive ambiguous, unpredictable, or insufficiently defined situations as aversive [20]. Contemporary models conceptualize intolerance of uncertainty as a transdiagnostic mechanism involved in fear of the unknown, anxiety, and related forms of psychopathology [Carleton, 2016]. In this framework, uncertainty may become a central source of threat, motivating attempts to reduce ambiguity and restore predictability." (pp.4)

R3.6

Throughout the authors mention 'Spearman correaltions', perhaps reword to 'non-parametric correlation (rho)'. People will know what the test is if you refer to it this way. Spearman was a eugenicist, as was other prominent statiticians such Pearson and Fisher. I don't think a lot of people are aware of this.

We thank the reviewer for this suggestion. We have revised the terminology throughout the manuscript and replaced references to "Spearman correlations" with "non-parametric correlations (rho)" as suggested. These changes can be found in the Methods and Results sections (pp. 10-12).

Reviewer #4:

This study aims to investigate the relationship between self-concept clarity, intolerance of uncertainty, and schizotypal traits, with a focus on whether intolerance of uncertainty moderates the association between self-concept clarity and positive schizotypy in a non-clinical context.

I've read it once before seeing the revised version and a second one after the revised version.

Thank you for evaluating our manuscript. We also appreciate the positive evaluation and the constructive points. Below, we reply one-by-one to your points, and attach the revised manuscript with track changes.

R4.1

Introduction:
-If the schizotypy is studied in a general population, it is worth adding non-clinical or 'in general population' in the title.

Thank you for the helpful suggestion. The title now reads: *The uncertain self: intolerance of uncertainty moderates the association of both positive and disorganized schizotypal traits with self-concept clarity in a non-clinical sample*

R4.2

- The introduction is going back and forth between self-clarity in schizophrenia, other psychiatric conditions, and the general population. It would be a good idea to concentrate then in the same paragraphs.

We thank the reviewer for this helpful suggestion. We agree that the original Introduction moved back and forth between findings from schizophrenia, other psychiatric conditions, and the general population, which may have reduced the clarity of the theoretical argument. We have revised the Introduction to improve its organization and now present the literature in a more structured sequence. Specifically, we first introduce schizotypy as a dimensional model of schizophrenia-spectrum liability, then discuss self-concept clarity in schizophrenia-spectrum phenomena, followed by evidence from non-clinical and broader psychopathology samples. We then introduce intolerance of uncertainty and its relevance to psychosis-spectrum traits before presenting the rationale for the present study. This revision was intended to make the transition from clinical schizophrenia research to non-clinical schizotypy and general-population mechanisms more coherent.

R4.3

- “These studies provide theoretical grounding for why intolerance of uncertainty may be a critical moderator. “

Why moderator? Why not mediation? I’m not saying that these studies provide theoretical grounding for why these variables may be a moderator, but authors should explain clearly why that is. What do these studies show in favor of a moderator? Why do these studies not show that intolerance of uncertainty may be a critical mediator? We need to understand the rationale behind this hypothesis. This is highly critical because the whole paper is based upon it. If it cannot be explained (which I’m sure it can), it might give the idea that this theoretical grounding has been written after seeing the statistical analysis.

We thank the reviewer for this important comment. We would like to clarify that the moderation hypothesis was specified a priori and was not formulated after inspecting the statistical results. However, we agree that the original manuscript did not explain this rationale clearly enough and that the cited studies do not directly establish intolerance of uncertainty as a moderator rather than a mediator. We have therefore revised the Introduction to make the theoretical and methodological rationale explicit.

Our rationale was that intolerance of uncertainty may shape the extent to which low self-concept clarity is associated with schizotypal traits. In other words, the focal question was not whether intolerance of uncertainty transmits the effect of self-concept clarity on schizotypy, but whether the self-concept clarity–schizotypy association differs depending on individuals’ tendency to experience uncertainty as aversive, threatening, or behaviorally disabling. This moderation framework is consistent with the conceptualization of intolerance of uncertainty as a relatively stable transdiagnostic vulnerability factor influencing responses to ambiguity, unpredictability, and perceived loss of control.

We also agree that a mediational pathway is theoretically plausible. However, testing mediation would require stronger assumptions about temporal ordering among self-concept clarity, intolerance of uncertainty, and schizotypal traits. Given the cross-sectional nature of the present data, we considered mediation less appropriate as the primary model. We now acknowledge this explicitly and identify longitudinal research as necessary to determine whether intolerance of uncertainty may also mediate associations between self-concept clarity and schizotypal traits.

R4.4

Methodology:

- I understand why you did not include lower-order subscales, but given the focus on the study on intolerance of uncertainty, it would be interesting to measure these subscales such

as ideas of reference, suspiciousness, magical thinking, and social anxiety. Maybe state this in the limitations?

Thank you for this helpful suggestion. We agree that lower-order schizotypy subscales may provide additional information in the context of intolerance of uncertainty. In the main analyses, we focused on the higher-order schizotypy dimensions because these are most commonly used in the literature and allowed for a more parsimonious model structure. However, in response to the reviewers' previous suggestions, we conducted supplementary analyses examining the relevant lower-order positive schizotypy subscales separately, including unusual experiences, ideas of reference, suspiciousness, and magical thinking. These analyses are now reported in the Supplementary Materials (pp. 22).

R4.5

- As I work in French, it is always difficult to find questionnaires validated in French. Could you please indicate to the reviewers and in the manuscript if you used all **questionnaires validated in Hungarian?** If it's the case, please report **chromback's alpha** for the validation study. If not, please indicate how you managed to use those.

Thank you for raising this important point. All questionnaires used in the present study had previously been validated in Hungarian. The original manuscript already included references to both the original instruments and their Hungarian adaptations; however, we have now clarified this more explicitly in the manuscript. We have also added the Cronbach's alpha values reported in the Hungarian validation studies where available (please see p. 9). Please see the reference details below.

SPQ-BR Hungarian adaptation: Kállai J, Rózsa S, Hupuczi E, Hargitai R, Birkás B, Hartung I, Martin L, Herold R, Simon M. Schizotypy Personality Questionnaire Brief Revisited (SPQ-BR) Hungarian adaptation and interpretation of factors. Psychiatr Hung. 2018;33(3):205–21. PMID: 30426928.

SCCS Hungarian adaptation: Hargitai R, Rózsa S, Hupuczi E, Birkás B, Hartung I, Hartungné E, Tiringer I, Martin L, Kállai J. Énkép egyértelműség mérése és korrelátumai. Magyar Pszichológiai Szemle. 2020;75(4). doi:10.1556/0016.2020.00033

IUS Hungarian adaptation: Zsidó NA, Arató N, Inhóf O, Budai T, Stecina TD, Lábadi B. A Bizonytalanságintolerancia Skála rövidített változatának magyar nyelvű adaptációja. Mentálhigiéné és Pszichoszomatika. 2021;22(1):103–20. doi:10.1556/0406.22.2021.003

In terms of the SPQ-BR, the Hungarian validation study provided Cronbach's alpha values for the seven first-order SPQ-BR subscales, but did not report separate reliability estimates

for the three higher-order schizotypy dimensions. We therefore refer to the validation study for the reliability of the subscales and report the internal consistency estimates for the positive, negative, and disorganized schizotypy dimensions as observed in the present sample.

R4.6

Statistical analysis:

- Please clarify what you mean by robust Spearman correlation. Do you mean the rank-based Spearman coefficient itself, because it is already relatively robust to outliers; or a robust test/variant of Spearman correlation, like using permutation methods or other adjustments? If it's the first one, please remove the word 'robust' and if it's the second one, please indicate which methods before the results, that is to say in the statistical analysis.

We thank the reviewer for pointing out this ambiguity. We did not use a separate robust variant or permutation-based test of the correlation coefficient; the term "robust" was intended only to refer to the rank-based nature of the correlation. To avoid confusion, we have removed the word "robust" and now refer to the analyses as "non-parametric correlations (rho)" throughout the manuscript. This change is reported in the Statistical analysis section and the Results section (pp. 10, 12, 13).

R4.7

- Have you please indicated whether you used a Holm or Bonferroni correction for your correlation matrix? Given the large effects, I believe you have not.

(after seeing the revised version: After Benjamini–Hochberg correction, all correlations remained significant. => Ok then you should use the **new p values in table 2.**).

Thank you for this comment. We have revised Table 2 to report the Benjamini–Hochberg corrected p-values for the correlation matrix. After correction, all correlations remained statistically significant. The level of statistical significance remained unchanged for all associations, with one exception: the association between self-concept clarity and the prospective dimension of intolerance of uncertainty changed from $p < .001$ to $p < .01$. This has now been corrected in Table 2 (pp. 13)..

R4.8

- Instead of linear regression, you might consider (for the next time) a general linear model in which you can scale the dependent variable on z-scores. Given the non-Gaussian distribution, this might be a good idea. But I'm not asking to redo your analysis.

We thank the reviewer for this helpful methodological suggestion. As the reviewer noted, we did not redo the analyses on this basis, but we agree that using a general linear modelling framework with standardized dependent variables may be useful in future studies, particularly when dealing with non-Gaussian distributions. We have taken this suggestion into consideration for future work.

R4.9

- The use of linear regression for moderator effect is okay. However, the whole thing is highly dependent on the predictors. Why did you use only the positive dimension of schizotypy as the dependent variable? As I understand why you controlled age and sex, I don't follow why you controlled for the disorganized dimension? I'm asking myself if we would find the moderator effect if you were not controlling the disorganized dimension. Negative SPQ is also significant with IUS PROS and IUS INH, so why did you not use it as a variable? If you used and found nothing, it should be also reported.

- What happens when you do not control for disorganized factors? If we lose the moderator effect, the whole thing gives the impression that the predictors are chosen after seeing the moderation effect.

(after seeing the revised version: I see you clarified all of the above. However, you got lucky to have the same moderation effect for positive schizotypy after changing controlled predictors. This is why I appreciate that you used OSF.

Thank you for this comment. We agree that the original analytic approach required clarification and that the selection of controlled predictors could otherwise give the impression of being data-driven. In the revised manuscript, following the suggestion of another reviewer, we re-ran the analyses using each of the three schizotypy dimensions as separate outcome variables. In each model, the other two schizotypy dimensions were entered as control variables. Thus, for example, when positive schizotypy was the outcome, negative and disorganized schizotypy were controlled for; when disorganized schizotypy was the outcome, positive and negative schizotypy were controlled for; and so forth. This approach allowed us to examine the specificity of the moderation effects across schizotypy dimensions while reducing concerns about selective predictor inclusion.

R4.10

Discussion:

- The paragraph in the limitations section regarding the demographics of the participants and Facebook is unnecessarily long. One phrase suffices.

Thank you for this suggestion. We have shortened the paragraph in the Limitations section concerning participant demographics and Facebook-based recruitment.

> "Although Facebook-based recruitment enabled access to a heterogeneous non-clinical sample, the overrepresentation of women indicates that the sample was not fully representative, which may limit the generalizability of the findings." (pp. 27)

---

7. PLOS authors have the option to publish the peer review history of their article (what does this mean?). If published, this will include your full peer review and any attached files.

If you choose "no", your identity will remain anonymous but your review may still be made public.

Do you want your identity to be public for this peer review? For information about this choice, including consent withdrawal, please see our Privacy Policy.

Reviewer #3: No

Reviewer #4: **Yes:** Ali Oker
