# Supplementary material for: The uncertain self: Intolerance of uncertainty moderates the association of both positive and disorganized schizotypal traits with self-concept clarity in a non-clinical sample
Source: PLoS One. 2026 Jul 2;21(7):e0352650. doi: 10.1371/journal.pone.0352650 (PMC13327182; doi:10.1371/journal.pone.0352650)
Supplement: Peer review history

# Peer review: Decision Letter 0

**DOI:** 10.1371/journal.pone.0352650.r001

**Authors:** Gerard Hutchinson

26 Nov 2025

PONE-D-25-52745

The uncertain self: intolerance of uncertainty moderates the association of positive schizotypal traits and self-concept clarity

PLOS ONE

Dear Dr. Kaszás,

Thank you for submitting your manuscript to PLOS ONE. After careful consideration, we feel that it has merit but does not fully meet PLOS ONE’s publication criteria as it currently stands. Therefore, we invite you to submit a revised version of the manuscript that addresses the points raised during the review process.

Please submit your revised manuscript by Jan 10 2026 11:59PM. If you will need more time than this to complete your revisions, please reply to this message or contact the journal office at plosone@plos.org. When you're ready to submit your revision, log on to https://www.editorialmanager.com/pone/ and select the 'Submissions Needing Revision' folder to locate your manuscript file.

Please include the following items when submitting your revised manuscript:

- A rebuttal letter that responds to each point raised by the academic editor and reviewer(s). You should upload this letter as a separate file labeled 'Response to Reviewers'.
- A marked-up copy of your manuscript that highlights changes made to the original version. You should upload this as a separate file labeled 'Revised Manuscript with Track Changes'.
- An unmarked version of your revised paper without tracked changes. You should upload this as a separate file labeled 'Manuscript'.

If you would like to make changes to your financial disclosure, please include your updated statement in your cover letter. Guidelines for resubmitting your figure files are available below the reviewer comments at the end of this letter.

If applicable, we recommend that you deposit your laboratory protocols in protocols.io to enhance the reproducibility of your results. Protocols.io assigns your protocol its own identifier (DOI) so that it can be cited independently in the future. For instructions see: https://journals.plos.org/plosone/s/submission-guidelines#loc-laboratory-protocols. Additionally, PLOS ONE offers an option for publishing peer-reviewed Lab Protocol articles, which describe protocols hosted on protocols.io. Read more information on sharing protocols at https://plos.org/protocols?utm_medium=editorial-email&utm_source=authorletters&utm_campaign=protocols.

We look forward to receiving your revised manuscript.

Kind regards,

Gerard Hutchinson, MD

Academic Editor

PLOS ONE

**Journal Requirements:**

When submitting your revision, we need you to address these additional requirements.

1. Please ensure that your manuscript meets PLOS ONE's style requirements, including those for file naming. The PLOS ONE style templates can be found at

https://journals.plos.org/plosone/s/file?id=wjVg/PLOSOne_formatting_sample_main_body.pdf and

https://journals.plos.org/plosone/s/file?id=ba62/PLOSOne_formatting_sample_title_authors_affiliations.pdf

2. We note that the grant information you provided in the ‘Funding Information’ and ‘Financial Disclosure’ sections do not match.

When you resubmit, please ensure that you provide the correct grant numbers for the awards you received for your study in the ‘Funding Information’ section.

3. Thank you for stating the following in the Acknowledgments Section of your manuscript:

“MR, ÁS and DB were supported by the NAP2022-I-2/2022 Research Grant (Hungarian Brain Research Program 3.0). BP and ÁS were supported by the Janos Bolyai Research Scholarship of the Hungarian Academy of Sciences.”

We note that you have provided funding information that is not currently declared in your Funding Statement. However, funding information should not appear in the Acknowledgments section or other areas of your manuscript. We will only publish funding information present in the Funding Statement section of the online submission form.

Please remove any funding-related text from the manuscript and let us know how you would like to update your Funding Statement. Currently, your Funding Statement reads as follows:

“MR, ÁS, and DB were supported by the NAP2022-I-2/2022 Research Grant

(Hungarian Brain Research Program 3.0). https://mta.hu

BP and ÁS were supported by the János Bolyai Research Scholarship of the

Hungarian Academy of Sciences. https://mta.hu/bolyai-osztondij/bolyai-janos-kutatasiosztondij-105319

The funders did not play any role in the study design, data collection and analysis,

decision to publish, or preparation of the manuscript.”

Please include your amended statements within your cover letter; we will change the online submission form on your behalf.

4. Please note that your Data Availability Statement is currently missing the repository name. If your manuscript is accepted for publication, you will be asked to provide these details on a very short timeline. We therefore suggest that you provide this information now, though we will not hold up the peer review process if you are unable.

5. If the reviewer comments include a recommendation to cite specific previously published works, please review and evaluate these publications to determine whether they are relevant and should be cited. There is no requirement to cite these works unless the editor has indicated otherwise.

**Additional Editor Comments:**

Both reviewers referred to a systematic review by Morriss et al, 2025, particular note should be made of these findings to incorporate into the manuscript. In addition, all of the points raised by the reviewers should be addressed

[Note: HTML markup is below. Please do not edit.]

Reviewers' comments:

Reviewer's Responses to Questions

**Comments to the Author**

1. Is the manuscript technically sound, and do the data support the conclusions?

The manuscript must describe a technically sound piece of scientific research with data that supports the conclusions. Experiments must have been conducted rigorously, with appropriate controls, replication, and sample sizes. The conclusions must be drawn appropriately based on the data presented.

Reviewer #1: Partly

Reviewer #2: Partly

**********

2. Has the statistical analysis been performed appropriately and rigorously?

Reviewer #1: Yes

Reviewer #2: Yes

**********

3. Have the authors made all data underlying the findings in their manuscript fully available?

The PLOS Data policy requires authors to make all data underlying the findings described in their manuscript fully available without restriction, with rare exception (please refer to the Data Availability Statement in the manuscript PDF file). The data should be provided as part of the manuscript or its supporting information, or deposited to a public repository. For example, in addition to summary statistics, the data points behind means, medians and variance measures should be available. If there are restrictions on publicly sharing data—e.g. participant privacy or use of data from a third party—those must be specified.

Reviewer #1: Yes

Reviewer #2: Yes

**********

4. Is the manuscript presented in an intelligible fashion and written in standard English?

PLOS ONE does not copyedit accepted manuscripts, so the language in submitted articles must be clear, correct, and unambiguous. Any typographical or grammatical errors should be corrected at revision, so please note any specific errors here.

Reviewer #1: Yes

Reviewer #2: Yes

**********

5. Review Comments to the Author

Please use the space provided to explain your answers to the questions above. You may also include additional comments for the author, including concerns about dual publication, research ethics, or publication ethics. (Please upload your review as an attachment if it exceeds 20,000 characters)

Reviewer #1: This manuscript “The uncertain self: intolerance of uncertainty moderates the association of positive schizotypal traits and self-concept clarity” reports moderation role of intolerance of uncertainty (IU) in relationship between self-concept clarity (SCC) and positive schizotypy in a sample of 315 normal adults. The manuscript is well written however it has concerns related to its objectives and methodology.

1. The authors’ decision to limit the study to the association between positive schizotypal personality and SCC needs further theoretical justification. Given that SCC and IU are reported to be related to all three schizotypal dimensions in the literature, as well as in the authors' own correlation data, the current justification—which primarily links IU to positive symptoms—does not fully explain the exclusion of the negative and disorganized dimensions from the primary analysis. Exploring the moderating role of IU across all three schizotypal dimensions would significantly enhance the theoretical breadth and informativeness of the manuscript.

2. The inclusion of only the disorganized dimension as a covariate in the regression equation predicting positive schizotypy lacks clear justification. For methodological consistency, the authors should consider either including both negative and disorganized dimensions as covariates or, alternatively, omitting both from the primary moderation analysis. Clarification on the rationale for the current choice is necessary.

3. The interpretation of the moderation effect, particularly the assumption of IU as an antecedent influencing the relationship between SCC and schizotypal personality requires stronger justification. Since both IU and SCC are stable personality-related constructs, establishing a clear antecedent-consequent relationship is difficult in a cross-sectional study. To support the current interpretation, the authors should cite longitudinal or experimental evidence showing IU as an antecedent of SCC. Failing this, the authors are encouraged to interpret the interactional relationship in both directions.

4. Given that the current results, alongside recent literature (e.g., Kumar et al., 2025; Morriss et al., 2025), demonstrate a direct relationship between IU and schizotypal personality, the authors should consider exploring a more complex model. A moderated mediation analysis might offer a richer understanding of the combined influence of IU. At minimum, the discussion of the moderation effects should also briefly address the potential for a mediating role of IU, which could lead to a more comprehensive theoretical explanation.

Reference

Kumar et al., 2025. The Role of Intolerance of Uncertainty and Religiousness in Schizotypal Personality and Life Satisfaction: A Cross-Sectional Study. The Journal of psychology, 159(2), 92–110.

Morriss et al. (2025). Intolerance of uncertainty and psychosis: A systematic review. The British journal of clinical psychology, 64(2), 344–354.

Reviewer #2: This study makes a valuable contribution to the literature by addressing the association between self-concept clarity, positive schizotypy and the effect of intolerance of uncertainty. The authors recruited a large non-clinical sample and assessed schizotypal traits (SPQ-BR), self-concept clarity (SCCS), and intolerance of uncertainty (IUS). Their correlational analyses indicated that lower self-concept clarity was negatively associated with higher levels across all schizotypy dimensions, as well as with both intolerance of uncertainty subscales. The subsequent moderation analyses further demonstrated that higher levels of intolerance of uncertainty attenuated the negative relationship between self-concept clarity and positive schizotypy. Findings suggest that intolerance of uncertainty may buffer, the extent to which reduced self-concept clarity relates to positive schizotypal traits.

1.Could the authors clarify whether they controlled for anxiety, or depressive symptoms in their study? Prior literature suggests that these factors are associated with both intolerance of uncertainty and self-concept clarity, and accounting for them could strengthen the interpretation of the findings.

2.Please report the specific inclusion and exclusion criteria used in participant recruitment. In particular, did the authors exclude individuals with current or past psychiatric diagnoses, and if so, how was this assessed? On page 16, the authors state that the study did not include individuals with a clinical diagnosis. Could the authors clarify how this was determined (e.g., screening questions, self-report, exclusion criteria)?

3. It would be helpful if the manuscript included the mean and standard deviation for each schizotypy dimension, the SCCS, and the IUS subscales.

4. A recent systematic review (Morriss et al., 2025) demonstrated stronger evidence linking intolerance of uncertainty to paranoia-related symptoms in nonclinical samples. In light of this, the authors may wish to conduct supplementary analyses focusing on SPQ subscales that most closely reflect paranoid ideation (e.g., suspiciousness, ideas of reference). This could offer additional insights into the specificity of the observed effects.

Morriss, J., Butler, D., & Ellett, L. (2025). Intolerance of uncertainty and psychosis: A systematic review. The British journal of clinical psychology, 64(2), 344–354. https://doi.org/10.1111/bjc.12509

5. The correlation analyses indicate significant associations between negative schizotypy, self-concept clarity, and intolerance of uncertainty. Could the authors elaborate on the rationale for not including negative schizotypy as a covariate in the moderation models?

6. On page 9, the authors state that “all associations were strongly negative.” However, the reported Spearman’s rho values (−0.386, −0.482, −0.528) fall within the moderate range. Please consider revising this description to more accurately reflect the magnitude of the correlations.

7. In Table 1, the correlation between prospective IUS and positive schizotypy is non-significant (p = .229). Could the authors provide an explanation or interpretation for this finding

8. Since the authors performed separate moderation analyses for the inhibitory and prospective IUS subscales, it would be beneficial to expand the Discussion section to address possible underpinnings of these two facets of intolerance of uncertainty

9. In the abstract, the authors state that inhibitory intolerance of uncertainty significantly moderated the relationship between self-concept clarity and positive schizotypy. However, according to the reported results, the prospective IU subscale also yielded a significant moderation effect. Please revise the abstract to reflect both findings and provide a balanced interpretation of the two moderation effects.

10. For future directions, the authors may consider discussing research on the interplay between childhood trauma, self-concept clarity, and psychopathology, as highlighted in prior work (e.g., Evans et al., 2015). This line of inquiry may provide additional context for understanding the developmental pathways underlying schizotypal traits.

11. There appears to be a minor typographical error in the references: ‘ref 255’ should be ‘ref 25’ page 5. Please correct this.”

**********

6. PLOS authors have the option to publish the peer review history of their article (what does this mean?). If published, this will include your full peer review and any attached files.

If you choose “no”, your identity will remain anonymous but your review may still be made public.

**Do you want your identity to be public for this peer review?**  For information about this choice, including consent withdrawal, please see our Privacy Policy.

Reviewer #1: No

Reviewer #2: No

**********

[NOTE: If reviewer comments were submitted as an attachment file, they will be attached to this email and accessible via the submission site. Please log into your account, locate the manuscript record, and check for the action link "View Attachments". If this link does not appear, there are no attachment files.]

To ensure your figures meet our technical requirements, please review our figure guidelines: https://journals.plos.org/plosone/s/figures

You may also use PLOS’s free figure tool, NAAS, to help you prepare publication quality figures: https://journals.plos.org/plosone/s/figures#loc-tools-for-figure-preparation.

NAAS will assess whether your figures meet our technical requirements by comparing each figure against our figure specifications.

# Author comment: Author response to Decision Letter 1

**DOI:** 10.1371/journal.pone.0352650.r002

27 Apr 2026

We have provided detailed responses to the reviewers’ comments in the uploaded file titled “Response to Reviewers.” Our responses describe how we addressed the issues and recommendations raised by the reviewers.

Attachment Submitted filename: Response to Reviewers.pdf

# Peer review: Decision Letter 1

**DOI:** 10.1371/journal.pone.0352650.r003

**Authors:** Gerard Hutchinson

19 May 2026

PONE-D-25-52745R1

The uncertain self: intolerance of uncertainty moderates the association of both positive and disorganized schizotypal traits and self-concept clarity

PLOS One

Dear Dr.  Kaszás,

Thank you for submitting your manuscript to PLOS ONE. After careful consideration, we feel that it has merit but does not fully meet PLOS ONE’s publication criteria as it currently stands. Therefore, we invite you to submit a revised version of the manuscript that addresses the points raised during the review process.

Please submit your revised manuscript by Jul 03 2026 11:59PM. If you will need more time than this to complete your revisions, please reply to this message or contact the journal office at plosone@plos.org. When you're ready to submit your revision, log on to https://www.editorialmanager.com/pone/ and select the 'Submissions Needing Revision' folder to locate your manuscript file.

Please include the following items when submitting your revised manuscript:

- A letter that responds to each point raised by the academic editor and reviewer(s). You should upload this letter as a separate file labeled 'Response to Reviewers'.
- A marked-up copy of your manuscript that highlights changes made to the original version. You should upload this as a separate file labeled 'Revised Manuscript with Track Changes'.
- An unmarked version of your revised paper without tracked changes. You should upload this as a separate file labeled 'Manuscript'.

If you would like to make changes to your financial disclosure, please include your updated statement in your cover letter. Guidelines for resubmitting your figure files are available below the reviewer comments at the end of this letter.

If applicable, we recommend that you deposit your laboratory protocols in protocols.io to enhance the reproducibility of your results. Protocols.io assigns your protocol its own identifier (DOI) so that it can be cited independently in the future. For instructions see: https://journals.plos.org/plosone/s/submission-guidelines#loc-laboratory-protocols. Additionally, PLOS ONE offers an option for publishing peer-reviewed Lab Protocol articles, which describe protocols hosted on protocols.io. Read more information on sharing protocols at https://plos.org/protocols?utm_medium=editorial-email&utm_source=authorletters&utm_campaign=protocols.

As the corresponding author, your ORCID iD is verified in the submission system and will appear in the published article. PLOS supports the use of ORCID, and we encourage all coauthors to register for an ORCID iD and use it as well. Please encourage your coauthors to verify their ORCID iD within the submission system before final acceptance, as unverified ORCID iDs will not appear in the published article. *Only*  the individual author can complete the verification step; PLOS staff *cannot*  verify ORCID iDs on behalf of authors.

We look forward to receiving your revised manuscript.

Kind regards,

Gerard Hutchinson, MD

Academic Editor

PLOS One

Journal Requirements:

1. If the reviewer comments include a recommendation to cite specific previously published works, please review and evaluate these publications to determine whether they are relevant and should be cited. There is no requirement to cite these works unless the editor has indicated otherwise.

2. Please review your reference list to ensure that it is complete and correct. If you have cited papers that have been retracted, please include the rationale for doing so in the manuscript text, or remove these references and replace them with relevant current references. Any changes to the reference list should be mentioned in the rebuttal letter that accompanies your revised manuscript. If you need to cite a retracted article, indicate the article’s retracted status in the References list and also include a citation and full reference for the retraction notice.

Additional Editor Comments:

Please address the comments made by the reviewers in your resubmission and indicate/highlight and explain the relevant changes.

[Note: HTML markup is below. Please do not edit.]

Reviewer's Responses to Questions

**Comments to the Author**

1. If the authors have adequately addressed your comments raised in a previous round of review and you feel that this manuscript is now acceptable for publication, you may indicate that here to bypass the “Comments to the Author” section, enter your conflict of interest statement in the “Confidential to Editor” section, and submit your "Accept" recommendation.

Reviewer #3: All comments have been addressed

Reviewer #4: All comments have been addressed

**********

2. Is the manuscript technically sound, and do the data support the conclusions?

The manuscript must describe a technically sound piece of scientific research with data that supports the conclusions. Experiments must have been conducted rigorously, with appropriate controls, replication, and sample sizes. The conclusions must be drawn appropriately based on the data presented.

Reviewer #3: Yes

Reviewer #4: Yes

**********

3. Has the statistical analysis been performed appropriately and rigorously?

Reviewer #3: Yes

Reviewer #4: I Don't Know

**********

4. Have the authors made all data underlying the findings in their manuscript fully available?

The PLOS Data policy requires authors to make all data underlying the findings described in their manuscript fully available without restriction, with rare exception (please refer to the Data Availability Statement in the manuscript PDF file). The data should be provided as part of the manuscript or its supporting information, or deposited to a public repository. For example, in addition to summary statistics, the data points behind means, medians and variance measures should be available. If there are restrictions on publicly sharing data—e.g. participant privacy or use of data from a third party—those must be specified.

Reviewer #3: Yes

Reviewer #4: Yes

**********

5. Is the manuscript presented in an intelligible fashion and written in standard English?

PLOS ONE does not copyedit accepted manuscripts, so the language in submitted articles must be clear, correct, and unambiguous. Any typographical or grammatical errors should be corrected at revision, so please note any specific errors here.

Reviewer #3: Yes

Reviewer #4: Yes

**********

6. Review Comments to the Author

Please use the space provided to explain your answers to the questions above. You may also include additional comments for the author, including concerns about dual publication, research ethics, or publication ethics. (Please upload your review as an attachment if it exceeds 20,000 characters)

Reviewer #3: Thank you for inviting me to review this interesting manuscript. I only have a few minor comments.

1. Can the authors refrain from using deficit based language throughout e.g.

'negative schizotypy reflects deficits in expected functions'

'reduced affective reactivity and motivational deficits'

'facilitate addressing self-related deficits'

'more vulnerable to the negative effects of failure'

'cognitive vulnerability'

The field is moving away from using such language as deficit and vulnerability. It is also potentially not accurate to use 'deficit' as there is nothing missing per se, but rather just differences in functioning. Regarding 'vulnerability', it is more appropriate to use the word 'risk'.

Dinishak, J. (2022). The deficit view and its critics. Disability Studies Quarterly, 36(4).

2. 'continuum between normality and schizophrenia' please don't use the word 'normality' when describing populations. What is normal? Perhaps, reword to 'schizoptpy exists on a conintuum and is more prevalent in schizophrenia.'

3. How do the authors know that their results are specific to IUS or SCCS. Did the authors measure any other broader negative affective traits (e.g. neuroticism) to control for? If not, please add this limitation and cite relevant papers addressing specificity of IU in relation to psychosis.

Morriss, J., & Ellett, L. (2025). Intolerance of uncertainty, paranoia, and prodromal symptoms: Comparisons between a schizophrenia spectrum disorder, anxiety disorder and non‐clinical sample. Psychology and Psychotherapy: Theory, Research and Practice, 98(4), 960-973.

Morriss, J., Gaudiano, B. A., So, S. H., Kingston, J., Lincoln, T., Morris, E. M., & Ellett, L. (2024). Associations between intolerance of uncertainty, paranoia, anxiety, and depression: Evidence from an international multisite sample. Mental Health Science, 2(4), e81.

4. The authors examine IU using the two dimensions. However,

-there is no rationale for using the two dimensions in the introduction/discussion

-the two dimensions are not mentioned in the hypotheses - even if exploratory

-report total IUS in the questionnaire section and its cronbach alpha please

-what happens when IUS total score is used in the models instead. Please report this in the correlation table as well for future meta-analyses. Given prior, research on IU and psychosis, I think the using the total score is warranted.

Hong, R. Y., & Lee, S. S. (2015). Further clarifying prospective and inhibitory intolerance of uncertainty: Factorial and construct validity of test scores from the Intolerance of Uncertainty Scale. Psychological assessment, 27(2), 605.

5. 'This anxiety can

contribute to intolerance of uncertainty, which refers to how individuals perceive and react to

ambiguous or unpredictable situations'. This section on IU could be expanded to included modern reviews and special issues on IU in relation to mechanisms:

Carleton, R. N. (2016). Fear of the unknown: One fear to rule them all?. Journal of anxiety disorders, 41, 5-21.

Morriss, J., Abend, R., Zika, O., Bradford, D. E., & Mertens, G. (2023). Neural and psychophysiological markers of intolerance of uncertainty. International Journal of Psychophysiology, 184, 94-99.

6. Throughout the authors mention 'Spearman correaltions', perhaps reword to 'non-parametric correlation (rho)'. People will know what the test is if you refer to it this way. Spearman was a eugenicist, as was other prominent statiticians such Pearson and Fisher. I don't think a lot of people are aware of this.

Reviewer #4: This study aims to investigate the relationship between self-concept clarity, intolerance of

uncertainty, and schizotypal traits, with a focus on whether intolerance of uncertainty

moderates the association between self-concept clarity and positive schizotypy in a non-clinical

context.

I’ve read it once before seeing the revised version and a second one after the revised version.

Introduction:

-If the schizotypy is studied in a general population, it is worth adding non-clinical or ‘in general population’ in the title.

- The introduction is going back and forth between self-clarity in schizophrenia, other psychiatric conditions, and the general population. It would be a good idea to concentrate then in the same paragraphs.

- “These studies provide theoretical grounding for why intolerance of uncertainty may be a critical moderator. “

Why moderator? Why not mediation? I’m not saying that these studies provide theoretical grounding for why these variables may be a moderator, but authors should explain clearly why that is. What do these studies show in favor of a moderator? Why do these studies not show that intolerance of uncertainty may be a critical mediator? We need to understand the rationale behind this hypothesis. This is highly critical because the whole paper is based upon it. If it cannot be explained (which I’m sure it can), it might give the idea that this theoretical grounding has been written after seeing the statistical analysis.

Methodology:

- I understand why you did not include lower-order subscales, but given the focus on the study on intolerance of uncertainty, it would be interesting to measure these subscales such as ideas of reference, suspiciousness, magical thinking, and social anxiety. Maybe state this in the limitations?

- As I work in French, it is always difficult to find questionnaires validated in French. Could you please indicate to the reviewers and in the manuscript if you used all questionnaires validated in Hungarian? If it’s the case, please report chromback’s alpha for the validation study. If not, please indicate how you managed to use those.

Statistical analysis:

- Please clarify what you mean by robust Spearman correlation. Do you mean the rank-based Spearman coefficient itself, because it is already relatively robust to outliers; or a robust test/variant of Spearman correlation, like using permutation methods or other adjustments? If it’s the first one, please remove the word ‘robust’ and if it’s the second one, please indicate which methods before the results, that is to say in the statistical analysis.

- Have you please indicated whether you used a Holm or Bonferroni correction for your correlation matrix? Given the large effects, I believe you have not.

(after seeing the revised version: After Benjamini–Hochberg correction, all correlations

remained significant. => Ok then you should use the new p values in table 2.).

- Instead of linear regression, you might consider (for the next time) a general linear model in which you can scale the dependent variable on z-scores. Given the non-Gaussian distribution, this might be a good idea. But I’m not asking to redo your analysis.

- The use of linear regression for moderator effect is okay. However, the whole thing is highly dependent on the predictors. Why did you use only the positive dimension of schizotypy as the dependent variable? As I understand why you controlled age and sex, I don’t follow why you controlled for the disorganized dimension? I’m asking myself if we would find the moderator effect if you were not controlling the disorganized dimension. Negative SPQ is also significant with IUS PROS and IUS INH, so why did you not use it as a variable? If you used and found nothing, it should be also reported.

- What happens when you do not control for disorganized factors? If we lose the moderator effect, the whole thing gives the impression that the predictors are chosen after seeing the moderation effect.

(after seeing the revised version: I see you clarified all of the above. However, you got lucky to have the same moderation effect for positive schizotypy after changing controlled predictors. This is why I appreciate that you used OSF.

Discussion:

- The paragraph in the limitations section regarding the demographics of the participants and Facebook is unnecessarily long. One phrase suffices.

**********

7. PLOS authors have the option to publish the peer review history of their article (what does this mean?). If published, this will include your full peer review and any attached files.

If you choose “no”, your identity will remain anonymous but your review may still be made public.

**Do you want your identity to be public for this peer review?**  For information about this choice, including consent withdrawal, please see our Privacy Policy.

Reviewer #3: No

Reviewer #4: **Yes:** Ali Oker

**********

[NOTE: If reviewer comments were submitted as an attachment file, they will be attached to this email and accessible via the submission site. Please log into your account, locate the manuscript record, and check for the action link "View Attachments". If this link does not appear, there are no attachment files.]

To ensure your figures meet our technical requirements, please review our figure guidelines: https://journals.plos.org/plosone/s/figures

You may also use PLOS’s free figure tool, NAAS, to help you prepare publication quality figures: https://journals.plos.org/plosone/s/figures#loc-tools-for-figure-preparation.

NAAS will assess whether your figures meet our technical requirements by comparing each figure against our figure specifications.

# Author comment: Author response to Decision Letter 2

**DOI:** 10.1371/journal.pone.0352650.r004

4 Jun 2026

We would like to thank the editor and reviewers for their thoughtful and constructive comments on our revised manuscript. We have addressed each comment in detail, and our point-by-point responses have been uploaded in the file entitled “Response to Reviewers - Revision 2.”

Attachment Submitted filename: Response to Reviewers - Revision 2.pdf

# Peer review: Decision Letter 2

**DOI:** 10.1371/journal.pone.0352650.r005

**Authors:** Gerard Hutchinson

15 Jun 2026

The uncertain self: intolerance of uncertainty moderates the association of both positive and disorganized schizotypal traits and self-concept clarity in a non-clinical sample

PONE-D-25-52745R2

Dear Dr. Kaszas

We’re pleased to inform you that your manuscript has been judged scientifically suitable for publication and will be formally accepted for publication once it meets all outstanding technical requirements.

Within one week, you’ll receive an e-mail detailing the required amendments. When these have been addressed, you’ll receive a formal acceptance letter and your manuscript will be scheduled for publication.

An invoice will be generated when your article is formally accepted. Please note, if your institution has a publishing partnership with PLOS and your article meets the relevant criteria, all or part of your publication costs will be covered. Please make sure your user information is up-to-date by logging into Editorial Manager at Editorial Manager® and clicking the ‘Update My Information' link at the top of the page. For questions related to billing, please contact billing support.

If your institution or institutions have a press office, please notify them about your upcoming paper to help maximize its impact. If they’ll be preparing press materials, please inform our press team as soon as possible -- no later than 48 hours after receiving the formal acceptance. Your manuscript will remain under strict press embargo until 2 pm Eastern Time on the date of publication. For more information, please contact onepress@plos.org.

Kind regards,

Gerard Hutchinson, MD

Academic Editor

PLOS One

Additional Editor Comments (optional):

Reviewers' comments:

Reviewer's Responses to Questions

**Comments to the Author**

1. If the authors have adequately addressed your comments raised in a previous round of review and you feel that this manuscript is now acceptable for publication, you may indicate that here to bypass the “Comments to the Author” section, enter your conflict of interest statement in the “Confidential to Editor” section, and submit your "Accept" recommendation.

Reviewer #3: All comments have been addressed

Reviewer #4: All comments have been addressed

**********

2. Is the manuscript technically sound, and do the data support the conclusions?

The manuscript must describe a technically sound piece of scientific research with data that supports the conclusions. Experiments must have been conducted rigorously, with appropriate controls, replication, and sample sizes. The conclusions must be drawn appropriately based on the data presented.

Reviewer #3: Yes

Reviewer #4: Yes

**********

3. Has the statistical analysis been performed appropriately and rigorously?

Reviewer #3: Yes

Reviewer #4: Yes

**********

4. Have the authors made all data underlying the findings in their manuscript fully available?

The PLOS Data policy requires authors to make all data underlying the findings described in their manuscript fully available without restriction, with rare exception (please refer to the Data Availability Statement in the manuscript PDF file). The data should be provided as part of the manuscript or its supporting information, or deposited to a public repository. For example, in addition to summary statistics, the data points behind means, medians and variance measures should be available. If there are restrictions on publicly sharing data—e.g. participant privacy or use of data from a third party—those must be specified.

Reviewer #3: Yes

Reviewer #4: Yes

**********

5. Is the manuscript presented in an intelligible fashion and written in standard English?

PLOS ONE does not copyedit accepted manuscripts, so the language in submitted articles must be clear, correct, and unambiguous. Any typographical or grammatical errors should be corrected at revision, so please note any specific errors here.

Reviewer #3: Yes

Reviewer #4: Yes

**********

6. Review Comments to the Author

Please use the space provided to explain your answers to the questions above. You may also include additional comments for the author, including concerns about dual publication, research ethics, or publication ethics. (Please upload your review as an attachment if it exceeds 20,000 characters)

Reviewer #3: (No Response)

Reviewer #4: My comments have been adressed. I found this version more interesting than the original one. Thank you for your collaboration.

**********

7. PLOS authors have the option to publish the peer review history of their article (what does this mean?). If published, this will include your full peer review and any attached files.

If you choose “no”, your identity will remain anonymous but your review may still be made public.

**Do you want your identity to be public for this peer review?**  For information about this choice, including consent withdrawal, please see our Privacy Policy.

Reviewer #3: No

Reviewer #4: **Yes:** Ali Oker (Université de Reims Champagne-Ardenne)

**********

# Editor report: Acceptance letter

**DOI:** 10.1371/journal.pone.0352650.r006

**Authors:** Gerard Hutchinson

PONE-D-25-52745R2

PLOS One

Dear Dr. Kaszás,

I'm pleased to inform you that your manuscript has been deemed suitable for publication in PLOS One. Congratulations! Your manuscript is now being handed over to our production team.

At this stage, our production department will prepare your paper for publication. This includes ensuring the following:

* All references, tables, and figures are properly cited

* All relevant supporting information is included in the manuscript submission,

* There are no issues that prevent the paper from being properly typeset

You will receive further instructions from the production team, including instructions on how to review your proof when it is ready. Please keep in mind that we are working through a large volume of accepted articles, so please give us a few days to review your paper and let you know the next and final steps.

Lastly, if your institution or institutions have a press office, please let them know about your upcoming paper now to help maximize its impact. If they'll be preparing press materials, please inform our press team within the next 48 hours. Your manuscript will remain under strict press embargo until 2 pm Eastern Time on the date of publication. For more information, please contact onepress@plos.org.

You will receive an invoice from PLOS for your publication fee after your manuscript has reached the completed accept phase. If you receive an email requesting payment before acceptance or for any other service, this may be a phishing scheme. Learn how to identify phishing emails and protect your accounts at https://explore.plos.org/phishing.

If we can help with anything else, please email us at customercare@plos.org.

Thank you for submitting your work to PLOS ONE and supporting open access.

Kind regards,

PLOS ONE Editorial Office Staff

on behalf of

Dr. Gerard Hutchinson

Academic Editor

PLOS One
